# Analysis of Deep Reinforcement Learning Algorithms for Task Offloading and Resource Allocation in Fog Computing Environments

**DOI:** 10.3390/s25175286

**Published:** 2025-08-25

**Authors:** Endris Mohammed Ali, Jemal Abawajy, Frezewd Lemma, Samira A. Baho

**Affiliations:** 1 Department of Computer Science and Engineering, College of Electrical Engineering and Computing, Adama Science and Technology University, Adama P.O. Box 1888, Ethiopia; endris.mohamed@astu.edu.et (E.M.A.); frezewd.lemma@astu.edu.et (F.L.); 2 Faculty of Science, Engineering and Built Environment, Deakin University, Geelong, VIC 3220, Australia; sbaho@deakin.edu.au

**Keywords:** deep reinforcement learning, fog computing, QoS, resource allocation, task offloading

## Abstract

Fog computing is increasingly preferred over cloud computing for processing tasks from Internet of Things (IoT) devices with limited resources. However, placing tasks and allocating resources in distributed and dynamic fog environments remains a major challenge, especially when trying to meet strict Quality of Service (QoS) requirements. Deep reinforcement learning (DRL) has emerged as a promising solution to these challenges, offering adaptive, data-driven decision-making in real-time and uncertain conditions. While several surveys have explored DRL in fog computing, most focus on traditional centralized offloading approaches or emphasize reinforcement learning (RL) with limited integration of deep learning. To address this gap, this paper presents a comprehensive and focused survey on the full-scale application of DRL to the task offloading problem in fog computing environments involving multiple user devices and multiple fog nodes. We systematically analyze and classify the literature based on architecture, resource allocation methods, QoS objectives, offloading topology and control, optimization strategies, DRL techniques used, and application scenarios. We also introduce a taxonomy of DRL-based task offloading models and highlight key challenges, open issues, and future research directions. This survey serves as a valuable resource for researchers by identifying unexplored areas and suggesting new directions for advancing DRL-based solutions in fog computing. For practitioners, it provides insights into selecting suitable DRL techniques and system designs to implement scalable, efficient, and QoS-aware fog computing applications in real-world environments.

## 1. Introduction

IoT devices, including consumer electronics (CE),TinyML-enabled systems, and unmanned aerial vehicles (UAVs), have seen widespread adoption across a range of industries. These network-connected devices are capable of sensing, processing, and communicating data. However, because they have limited computing power, they often need to offload tasks to more powerful systems to meet QoS requirements [1]. Task offloading refers to the process of transferring computational workloads from resource-limited devices to more powerful computing systems, based on a machine-to-machine collaboration model [2,3].

Traditionally, IoT devices offload tasks to cloud environments. However, device-to-cloud offloading introduces significant latency and bandwidth overhead, as well as potential privacy concerns [4], making it unsuitable for delay-sensitive applications. To overcome these limitations, fog computing was introduced as a decentralized approach that brings computational resources closer to edge devices, allowing localized task execution and storage [5]. This proximity significantly reduces latency and enhances real-time processing efficiency [6]. By handling tasks near the data source, fog computing also improves the responsiveness of latency-sensitive applications and optimizes both communication and computation costs in line with QoS requirements [7].

Task offloading and resource allocation in distributed fog computing environments are inherently challenging due to the loosely coupled, highly dynamic, heterogeneous, and failure-prone nature of fog nodes [8]. In contrast to centralized cloud systems that operate within controlled and uniform infrastructures, fog computing must orchestrate diverse, geographically distributed resources while adhering to stringent QoS requirements. This complexity is further compounded by variable resource availability, user mobility, and the diverse performance needs of latency-sensitive applications. Achieving minimal communication and computation delays is crucial for enabling real-time analytics and mission-critical IoT operations. Moreover, the decentralized nature of fog computing introduces heightened concerns around data security, privacy protection, and effective data management, all of which are essential for maintaining trust and ensuring regulatory compliance [9]. Overcoming these challenges requires the development of intelligent, adaptive, and resource-aware task offloading strategies capable of optimizing performance while ensuring resilience against failures and accommodating heterogeneity in both hardware and network environments.

DRL has gained significant traction as a promising approach for addressing the complex challenges of task offloading and resource allocation in fog computing environments [10]. By enabling adaptive, real-time decision-making under uncertain and dynamic conditions, DRL offers considerable potential to enhance the efficiency and responsiveness of fog-based systems. The increasing application of DRL in this domain highlights the need for a comprehensive and systematic review of state-of-the-art DRL-based task offloading techniques, with particular attention to their role within decentralized and latency-sensitive fog computing environments. Although several surveys have investigated task offloading in fog computing, most existing reviews primarily highlights conventional ML [11], RL [12], and DL [1] approaches. While DRL has recently gained attention, its application is usually limited to general resource management [13] or computation offloading in edge-centric architectures [14]. Moreover, much of the literature still emphasizes centralized edge or mobile edge computing (MEC) models, overlooking the decentralized, heterogeneous, and latency-sensitive characteristics that define fog computing environments [1]. This highlights the absence of a dedicated and systematic survey that reviews and synthesizes the application of DRL for task offloading and resource allocation in fog computing environments. Consequently, there is a clear need for a focused review that consolidates recent developments, categorizes DRL-based techniques, and identifies open challenges and future research directions in fog-based systems.

This study addresses the aforementioned gap by exploring how DRL is used in many-to-many task offloading and resource allocation between IoT devices and fog nodes, with a focus on real-time, latency-sensitive applications. We present a comprehensive literature survey on task offloading across different layers, including IoT devices, edge, and fog nodes, as well as inter-fog node offloading. A key focus of this survey is the exploration of DRL-based offloading approaches and the growing role of smart devices in enabling real-time service provisioning. The study proposes a conceptual task offloading model and a supporting offloading architecture and examines how emerging technologies can be leveraged to meet QoS requirements for latency-sensitive applications. It also highlights the potential of knowledge-driven DRL frameworks in intelligent systems. These studies often overlook challenges related to continuous action spaces, convergence, and real-time adaptation. The main contributions of this survey are summarized as follows:

- Comprehensive Analysis of Task Offloading and Resource Allocation: We investigate to provide detailed knowledge about task offloading strategies and resource allocation mechanisms in fog computing, outlining core concepts, standards, and techniques to meet application-level QoS requirements.
- Topological Models and Control Architectures: We examine task offloading topology models and their patterns, focusing on resource management and control architectures across mixed, mobile, and fixed device-fog interaction categories.
- DRL-Based Approaches in Fog Environments: We explore the application of DRL for task offloading and resource allocation in dynamic fog and edge environments, highlighting current practices and future opportunities to enhance intelligent, adaptive systems.
- Real-World Use Cases and Emerging Strategies: Through a synthesis of cutting-edge research and practical applications, we demonstrate how DRL-based offloading can drive innovation in smart device and fog resource management.
- Research Gaps and Future Directions: We identify open challenges and future research directions in DRL-based task offloading within the fog computing paradigm.

The remainder of this paper is organized as follows: Section 2 provides background information and reviews related work. Section 3 outlines the research methodology. Section 4 presents the findings and the analysis. Section 5 discusses the DRL-based task offloading architecture in fog computing environments. The subsections of (Section 5) also extend the discussion with the details of DL, RL, and DRL taxonomy, DRL algorithms, task execution models, and applications of DRL-based task offloading in fog computing. Section 6 discusses DRL approaches integrated with QoS optimization strategies. Section 7 highlights future research directions and open issues. Section 8 concludes the paper.

## 2. Background and Related Work

In this section, we present a four-layered task offloading architecture, followed by a comprehensive review of related work.

### 2.1. Device-Edge-Fog-Cloud Architecture

**Figure 1:**
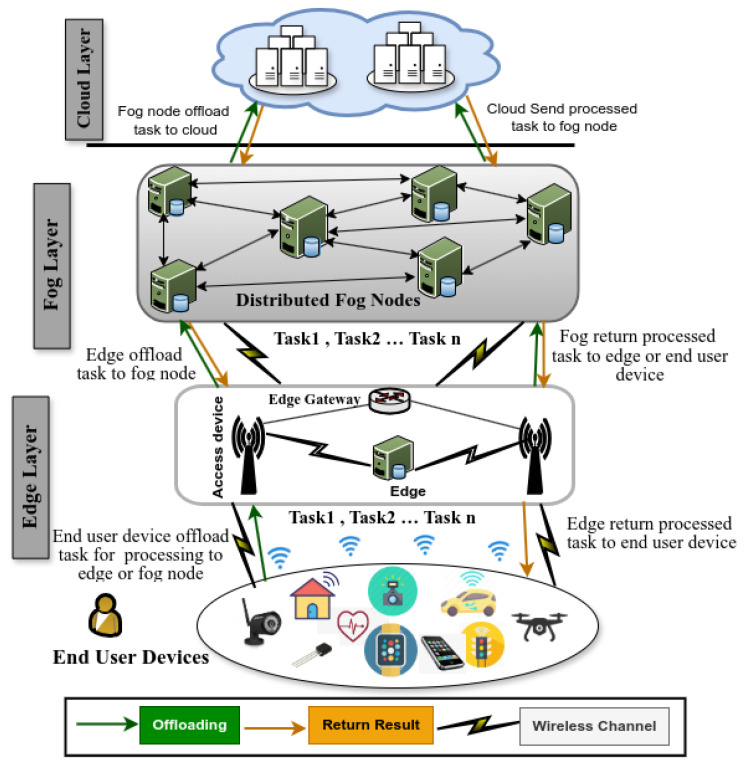
Edge–fog–cloud continuum architecture.

Figure 1 shows a high-level task offloading architecture consisting of four layers: end-user devices, edge, fog, and cloud. The first layer includes various end-user devices with limited computing power. The second layer consists of edge nodes that offer immediate, low-latency support for task offloading and often operate closely with the end-user devices. The third layer, the primary focus of this article, is the fog layer, which provides distributed, intermediate computing resources located near the data source. Finally, the fourth layer comprises centralized cloud data centers, suited for large-scale processing tasks that are not time-sensitive.

Edge computing is a distributed computing platform in which computation, storage, and networking resources are deployed at or near the network’s edge—close to where data are generated. This infrastructure can include gateways, base stations, or local edge servers, enabling low-latency processing without relying solely on centralized cloud resources [15]. This proximity-based model, illustrated in Figure 1, shows access nodes operating parallel to the end-user device. Edge computing is inherently device-centric and localized, with performance highly dependent on the strategic placement of edge servers—particularly in dynamic platforms like the Internet of Vehicles (IoV) [16].

In contrast, fog computing introduces an additional intermediate layer between edge devices and the cloud, comprising a distributed network of fog nodes capable of more complex, scalable, and context-aware processing tasks [4]. It adopts a collaborative and horizontally scalable architecture, enabling multiple nodes to share resources, orchestrate services, and perform real-time analytics across wider geographic areas. This makes it especially well-suited for large-scale, latency-sensitive applications, such as smart transportation and industrial IoT, that require rapid decision-making without relying solely on centralized cloud infrastructure.

In summary, fog computing and edge computing share the goal of bringing computation closer to data sources to reduce latency and bandwidth usage, yet they differ in architecture, scope, management, scalability, security, and functionality [15]. Singh et al. [17] and Laroui et al. [18] note that both paradigms follow distributed architectural principles but characterize fog computing as decentralized, flat, and hierarchical, with horizontal node collaboration enabling scalable and flexible service deployment.

### 2.2. Previous Surveys

Task offloading has emerged as a significant research focus in the context of fog computing, and numerous survey studies have explored this topic in depth. Table 1 presents a consolidated overview of key surveys related to fog-based task offloading, summarizing the various problem-solving approaches adopted in each work.

Lahmar and Boukadi [19] focus on heuristic, meta-heuristic, and exact matching optimization techniques for resource allocation in fog computing, aiming to enhance QoS for latency-sensitive applications. Hong and Varghese [20] provide a comprehensive classification framework encompassing fog computing architectures, infrastructures, and underlying algorithms. Similarly, Salaht et al. [21] examine the challenges of service placement and resource management in both edge and fog computing environments. Kar et al. [22] investigate task offloading in federated systems, offering a comparative classification of edge, fog, and cloud computing layers, along with a roadmap of offloading strategies tailored to different federated scenarios. Finally, Mukherjee et al. [23] discuss the use of cloudlets for mobile offloading, addressing both mobile and non-mobile application contexts. Most of these studies employ conventional centralized approaches for task offloading and resource allocation, where multiple smart user devices offload tasks to a central edge node for decision-making. While this model simplifies coordination, it limits the flexibility and scalability of task offloading in distributed fog computing environments.

**Table 1:** Summary of related surveys.

Reference	Approaches	Focus	Architectural Scope
[24]	Distributed ML (DML)	Distributed computation	IoT–Edge computing
[6]	ML	Resource allocation	Fog computing
[13]	DRL	Resource management	IoT–Fog computing
[14]	DRL	Computation offloading	Edge computing
[19]	ML	Resource allocation	Fog-to-Cloud and Fog-to-Fog
[25]	ML	Communication and computation	IoT–Edge-to-Cloud computing
[26]	ML, RL	Mobile-based computation offloading	Smart user device–MEC
[11]	ML	Resource management	IoT–Fog
[22]	Traditional/ML	Task offloading in federated system	Edge–Cloud and Fog–Fog
[27]	FRL	Offloading performance in RL	IoT–Edge–Fog–Cloud
[12]	RL	Resource allocation for task offloading	IoT–Fog–Cloud
[1]	RL	Computation offloading	IoT–Edge
[28]	ML	Computation offloading	Edge and Fog
Our Work	DRL	Task offloading and resource allocation	IoT–Edge–Fog

Salaht et al. [21] conducted a comprehensive survey on the taxonomy of service placement problems, focusing on two primary scenarios: (i) assigning services in a way that satisfies system requirements while optimizing a specific objective, and (ii) minimizing latency while ensuring acceptable QoS during service deployment. Their work primarily addresses the critical question: “Where should a service be deployed and executed to best meet QoS objectives?” Building on this foundation, the current survey extends the discussion by exploring DRL approaches for task offloading within smart computing environments—specifically between smart user devices, the edge, and fog architecture. This shift in perspective emphasizes the dynamic and adaptive nature of DRL in supporting real-time service demands and complex offloading decisions. To support the development of a DRL-based task offloading and resource allocation framework, the following subsections present the task computation model and relevant application scenarios in detail, forming the basis for modeling intelligent offloading strategies in distributed Fog computing environments.

The potential of DRL to enhance task offloading efficiency across various architectural settings has been recognized. Fahimullah et al. [11] examined different machine learning (ML) approaches for resource management in dynamic fog computing environments. Their findings indicate that Q-learning and DRL are among the most commonly applied techniques; however, their work does not focus specifically on task offloading. Among the studies that do target offloading, Shakarami et al. [29] provide a comprehensive review of computation offloading in IoT–MEC ecosystems using supervised, unsupervised, and RL methods. Similarly, Rodrigues et al. [25] explore ML techniques while highlighting the challenges posed by high-dimensional data in making offloading decisions. However, both studies do not address the dynamic status of resource availability or the inter-server communication required for real-time updates in distributed environments. To address this, Hortelano et al. [1] apply RL techniques to the task offloading problem in IoT–Edge networks. Nonetheless, their proposed architecture primarily follows a many-to-one offloading model, where multiple user devices offload tasks to a single edge node—limiting its application in fully distributed fog architectures. Further advancing this field, Tran-Dang et al. [12] present a survey that highlights the use of reinforcement learning for fog resource allocation, aiming to optimize task execution performance. However, their review also reveals that a key challenge still remains: Effectively deciding where to offload tasks in dynamic and distributed fog environments is yet to be fully addressed.

Several studies have surveyed fog computing from a range of perspectives, including resource allocation, infrastructure, algorithms, architecture, management models, and computation offloading [19,22,30,31]. While many of these works explore task offloading in fog environments, there is still limited focus on the opportunities and challenges presented by DL, RL, DRL, and federated learning (FL), particularly in the context of distributed fog node architectures. These gaps highlight the need for further investigation into adaptive task offloading strategies capable of operating in dynamic and heterogeneous fog computing ecosystems. To address this, the current study explores the application of AI/ML techniques, specifically, DL, RL, and DRL, for many-to-many task offloading and resource allocation. The scenario considered involves multiple smart user devices offloading tasks to multiple distributed fog nodes, aiming to meet the demands of real-time, latency-critical applications. In particular, the study focuses on how DRL can improve offloading decisions and optimize resource utilization between user devices and fog environments. It also provides insights into knowledge-driven multi-agent DRL approaches and their potential applications in emerging intelligent systems.

## 3. Research Methodology and Materials

This section explains the methodology used in conducting the study. The structure of the survey, including the research questions, keyword selection, and literature search strategy, is detailed in the following subsections.

### 3.1. Methodology

**Figure 2:**
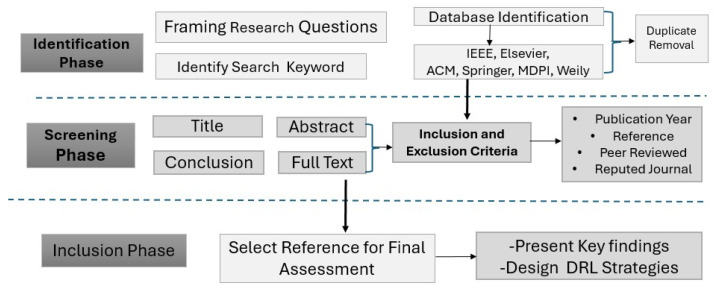
Steps of a comprehensive literature review process [33].

This survey covers research from 2017 to June 2025 on recent advances and emerging trends in DRL-based task offloading and resource allocation in fog computing. To ensure a rigorous and reproducible review process, we adopted a systematic methodology based on the widely recognized guidelines proposed by Kitchenham et al. [32] for conducting systematic literature reviews in software engineering. This structured approach enables the identification, evaluation, and synthesis of relevant studies in a transparent and methodical manner. As illustrated in Figure 2, the methodology is organized into three main phases, each designed to support a clear progression from research question formulation to literature selection, data extraction, and synthesis of the findings [33].

### 3.2. Formulation of Research Questions

We began by formulating key research questions aimed at exploring DRL-based task offloading approaches across various scenarios relevant to business applications. These foundational questions were carefully constructed to guide the scope of the literature review, identify knowledge gaps, and examine how DRL can be applied to optimize offloading decisions, improve resource allocation, and meet performance requirements in dynamic and distributed fog computing environments. Table 2 presents the research questions alongside their underlying motivations, helping to narrow the focus of the study.

### 3.3. Study Selection

**Figure 3:**
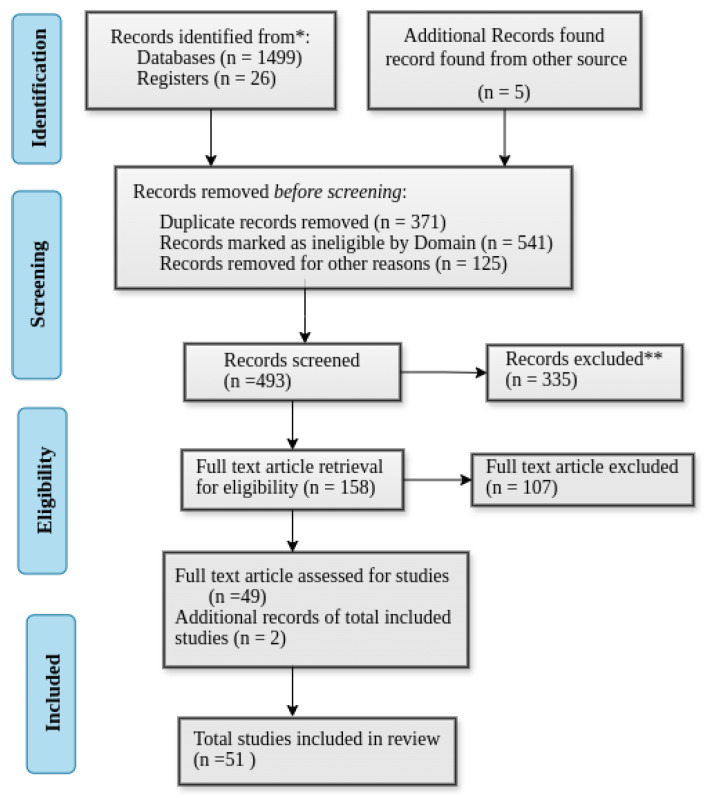
Paper selection flow diagram (* indicates the number of records identified from each database and register, and ** indicates the number of records excluded by automation tools, and manually by human review).

We used the PRISMA 2020 framework depicted in Figure 3 that shows the flow diagram for the paper organization process collected from the research database and other sources. It shows every step followed to organize our records.

#### 3.3.1. Eligibility Criteria

The inclusion criteria encompass research focused on task offloading and resource allocation between user devices, edge computing, and fog computing. Eligible works include articles published in peer-reviewed journals that address task offloading problems using DL, RL, and RL techniques. The review also considers studies that propose and evaluate ML-based approaches for task offloading and resource allocation in end-user device–fog computing scenarios. Furthermore, selected research must examine performance metrics such as energy efficiency, latency, throughput, resource utilization, and channel allocation. Only full-text articles published in journals indexed in the Web of Science, Scopus, or Science Citation Index (SCI) databases are included.

The exclusion criteria eliminate studies that do not specifically address DRL-based task offloading and resource allocation, including those primarily focused on unrelated IoT–fog topics. Non-peer-reviewed materials such as book chapters, conference abstracts, and other grey literature are excluded. Papers lacking sufficient methodological detail, such as case reports without clear procedures, are also omitted. Additionally, studies that do not involve task offloading between the user device and the fog layer—such as those focusing solely on cloud computing or edge computing without considering the fog—are excluded.

#### 3.3.2. Search Strategy and Data Collection Process

We use AND (&&) and OR (||) to search articles in scientific databases. After a regress study, the search query is presented as follows: [(“Task offloading” OR “Resource allocation” OR “Machine learning” OR “DL and RL-based task offloading”, OR “DRL based task offloading”) AND (“fog computing” OR “fog nodes” OR “distributed computations”)] were used as search keywords to include research works.

We designed a data extraction form based on QoS metrics such as energy consumption, latency, throughput, resource utilization, and fault tolerance to collect information from articles. Additionally, to capture relevant information about included studies, the data extraction form considers the year of publication, journal impact factors, study design, fog architecture, ML methods and algorithms, task characteristics, and evaluation methodology.

### 3.4. Synthesis of Common DRL Workflow

**Figure 4:**
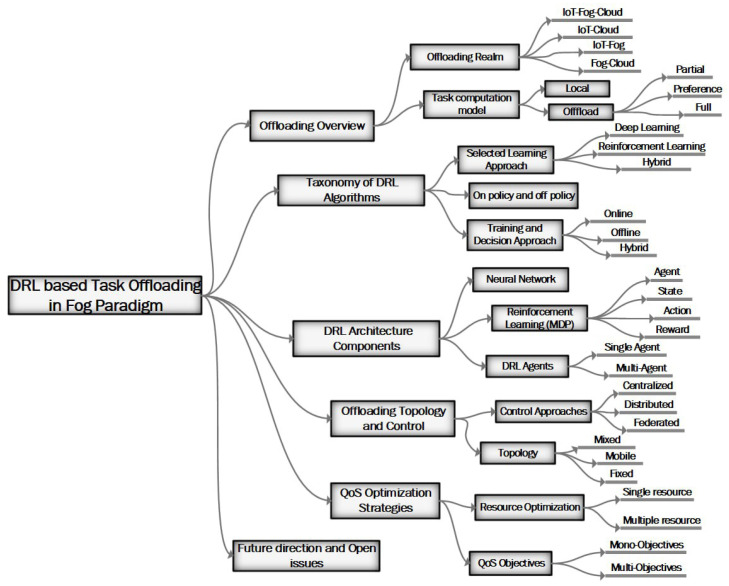
Overall survey structure.

While the primary focus of this survey is on summarizing existing works, we provide in Algorithm 1 a generalized pseudo-code that synthesizes common DRL-based task offloading processes from the literature [34,35,36,37,38]. This pseudo-code is not a novel algorithm but an illustrative abstraction representing the workflow typically reported across reviewed studies. Figure 4 presents the detailed literature survey plan, structured as a taxonomy framework that categorizes key aspects of task offloading and execution in the fog computing paradigm.
**Algorithm 1:** Generalized DRL Workflow for Task Offloading in Fog Computing
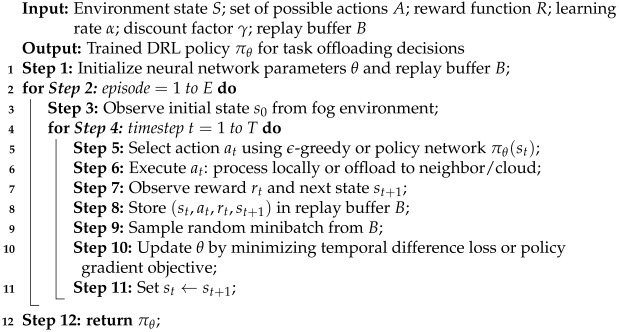


## 4. Findings and Analysis

This section presents the key findings derived from the analysis of selected studies and evaluates their implications within the context of DRL-based task offloading and resource allocation in fog computing environments. This section interprets the outcomes in relation to the research objectives, highlighting performance trends, the comparative strengths and weaknesses of existing approaches, and their alignment with identified evaluation metrics. Furthermore, it explores how the results contribute to advancing the field, addresses potential limitations, and discusses the practical relevance of the findings in real-world fog computing scenarios.

### 4.1. Trend Analysis

**Figure 5:**
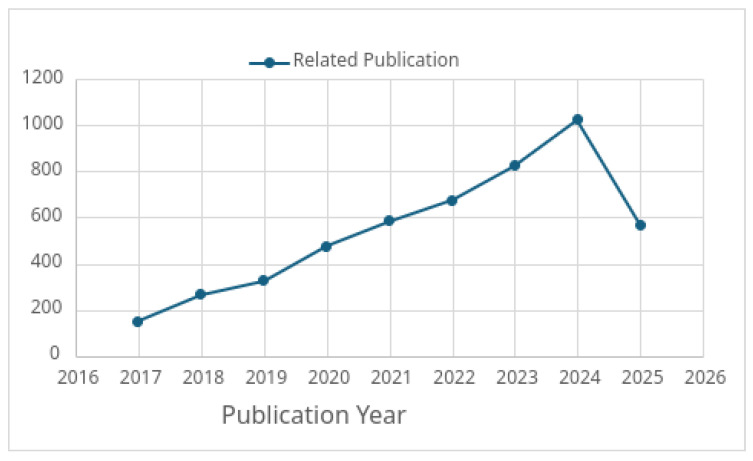
Distribution of the publications from 2017 to 2025.

According to the Web of Science (WoS) research trend analysis tool, the graph presented in Figure 5 highlights the growing importance and the increasing volume of research focused on fog computing integrated with machine learning (ML) techniques to address task offloading and resource allocation challenges. Notably, advanced methods such as deep learning DL, RL, DRL, and hybrid approaches are increasingly being applied to support real-time application requirements in dynamic environments.

**Figure 6:**
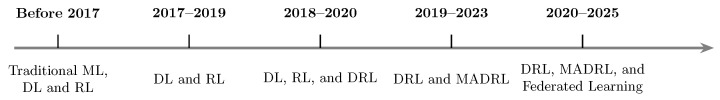
Timeline of learning algorithms for task offloading and resource allocation in fog computing environments.

Figure 6 illustrates the progression of the learning algorithms used in the computation offloading and resource allocation in fog computing environments. Initially, traditional ML, DL, and RL techniques were applied in centralized device-to-cloud offloading models. This was followed by the adoption of decentralized offloading in device-to-edge-to-cloud systems, device-to-MEC, and device–fog–cloud, and the emergence of MADRL for multi-UAV and multi-agent fog coordination. More recently, the field has shifted toward decentralized and federated MADRL in fog and edge environments.

### 4.2. Bibliometric Analysis

**Figure 7:**
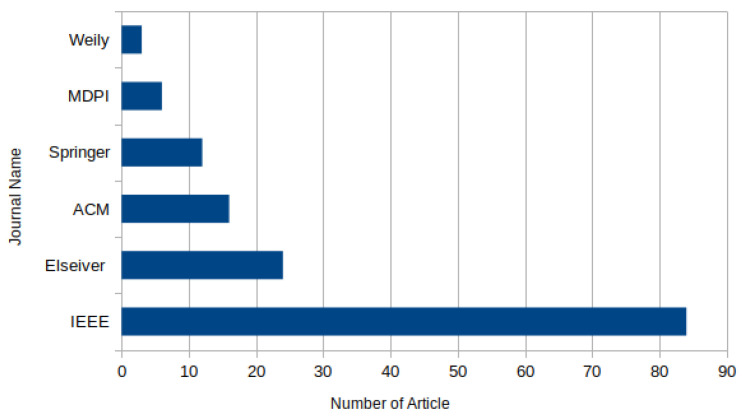
Journal database.

Figure 7 illustrates the distribution of published articles on DRL-based task offloading in fog computing across major academic journals and publishers. To deepen the bibliometric analysis of DRL applications in fog computing, incorporating publication year trends and the distribution between conference and journal publications offers valuable context into how this field has evolved. Particularly after 2018, there has been a noticeable rising trend in research activity over the past decade. This rise follows the maturation of fog computing as a fundamental infrastructure for low-latency and distributed IoT services and the more widespread use of DRL methods in several disciplines.

Between 2016 and 2018, research on fog-based resource management mostly used either DL alone or conventional RL. Starting in 2019, though, DRL-specific publications grew dramatically in numbers. The increasing awareness that DRL was well adapted to handle the complexity and movement of fog surroundings through its combination of perception (DL) and sequential decision-making (RL) drove this shift. More sophisticated paradigms including multi-agent DRL (MADRL) and federated DRL approaches started to be researched in 2022, therefore tackling scalability and decentralization more efficiently. These patterns imply that the field is maturing in terms of methodological complexity and practical relevance as well as increasing in volume. Regarding distribution channels, conference and journal publications exhibit a striking divide.

Regarding dissemination channels, there is a significant divide between conference and journal publications. Conference proceedings account for the majority, approximately 60%, of the research output, with IEEE-sponsored venues such as ICC, INFOCOM, and Globecom being the most prominent. These platforms are particularly preferred for the rapid dissemination of novel ideas and early-stage prototypes, often showcasing new DRL-based algorithms or architectures for task offloading. Journal publications, on the other hand—which make up roughly 40% of the literature—usually show up in high-impact venues like IEEE Access, ACM Transactions, and Elsevier’s Future Generation Computer Systems. These works tend to provide more comprehensive evaluations, real-world use cases, and longitudinal performance assessments, often addressing challenges like energy efficiency, service latency, and resource utilization in depth.

In summary, the bibliometric trends in DRL-based fog computing research reflect both growing interest and advancing maturity. The field’s relevance is underlined by the growing number of publications year over year; the balance between conference and journal outputs indicates a healthy research pipeline—from early invention to applied validation. These insights highlight how DRL has developed from an emerging technology into a strategic tool with emphasis on managing complex and distributed fog computing systems.

### 4.3. Empirical Distribution of DRL Algorithms

**Figure 8:**
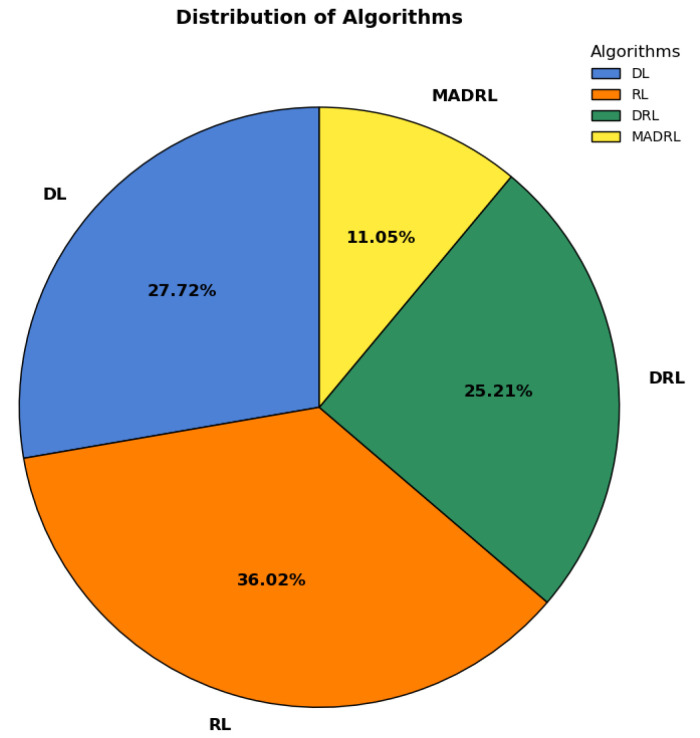
Research work distribution using DL, RL, DRL, and MADRL algorithms.

This section presents a comparative analysis of DRL in relation to DL and RL, followed by a discussion on the empirical distribution of various DRL algorithms. The pie chart in Figure 8 extracted from our database shows that RL techniques are the most commonly used approach by 36.02% to solve the joint task offloading and resource allocation decision problem, followed by DL 27.72%, DRL 25.21%, and MADRL 11.05%. This shows that DRL with the MADRL approach accounts for 36.26%, indicating a substantial increase in the adoption of DRL algorithms for task offloading and resource allocation in fog environments.

**Figure 9:**
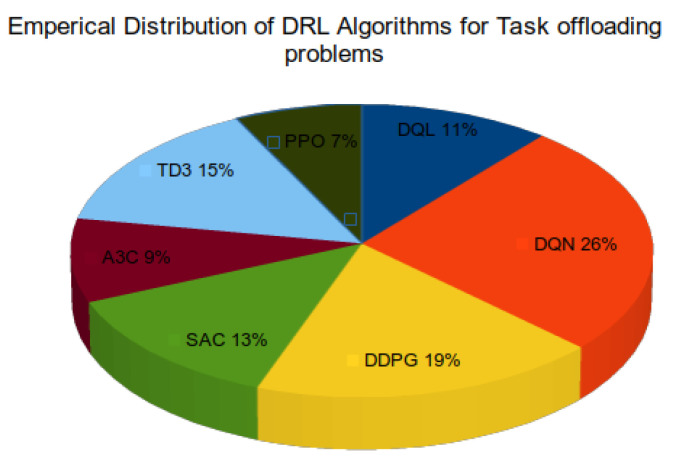
Empirical distribution of DRL algorithms.

Figure 9 illustrates the empirical distribution of various DRL algorithms used to address task offloading problems in fog computing environments. Among the surveyed studies, Deep Q-Network (DQN) emerges as the most widely adopted algorithm, accounting for 26% of the implementations. DQN’s popularity can be attributed to its relatively simple architecture and strong performance in discrete action spaces, making it well suited for decision-making tasks like binary offloading or server selection.

Deep Deterministic Policy Gradient (DDPG), with a 19% share, follows DQN to have the second-highest share. It is particularly successful in continuous action spaces—which are prevalent in fog computing situations requiring fine-grained resource allocation decisions. Then follow, with 15% and 13%, respectively, Twin Delayed Deep Deterministic Policy Gradient (TD3) and Soft Actor–Critic (SAC), showing rising interest in off-policy actor–critic techniques offering more steady training and better exploration. More current research, however, emphasizes a rising inclination toward the TD3 algorithm because of its better performance across several aspects. Faster convergence, lower latency, lower energy consumption, and better cooperative decision-making in distributed settings are all features of TD3 emphasized by studies such as Wakgra et al. [37], Ali et al. [38], Wakgra et al. [39], and Chen et al. [40]. Furthermore, as emphasized by Tadele et al. [41] and Ali et al. [38], TD3 effectively handles large action spaces and supports decentralized decision-making. With clipped double-Q learning, delayed policy updates, and target policy smoothing, all of which help for more stable training and reduced overestimation bias, TD3’s architecture is enhanced compared to earlier algorithms. These characteristics make TD3 exceptionally suited for complicated, dynamic, and decentralized fog computing settings, especially when used implemented in multi-agent systems.

The Advantage Actor–Critic (A3C) algorithm accounts for 9%, indicating moderate usage due to its ability to handle asynchronous updates and parallel agents, which align well with distributed fog environments. Deep Q-Learning (DQL), a classic variant of DQN, is used in 11% of the studies, while Proximal Policy Optimization (PPO) comprises the smallest share at 7%, despite its robust policy optimization characteristics.

Overall, the distribution highlights a clear preference for value-based techniques such DQN, as well as growing acceptance of hybrid actor–critic systems like DDPG and TD3. These algorithms, well suited for task offloading in dynamic and resource-constrained fog computing situations, strike a pragmatic balance between decision-making performance and implementation complexity. Notably, recent research shows a clear shift from conventional value-based approaches toward more sophisticated actor–critic methods, including SAC and TD3. This transition shows the increasing need for more flexibility, training stability, and scalability to successfully handle the heterogeneous and distributed nature of contemporary fog-based systems.

### 4.4. Performance Metrics

**Figure 10:**
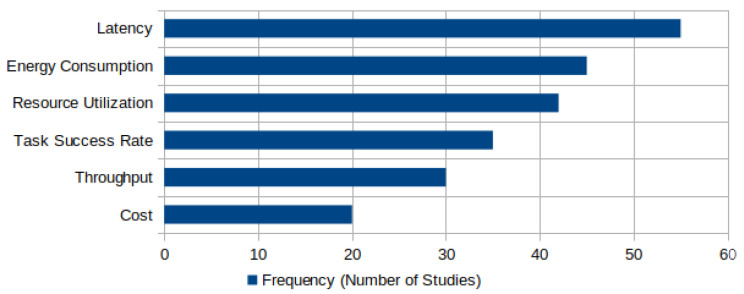
Key performance metrics used in DRL-based task offloading studies in fog computing environments.

Figure 10 summarizes the key performance metrics commonly used in DRL-based task offloading studies within fog computing environments. The *x*-axis represents the frequency, i.e., the number of studies that use each metric, while the *y*-axis lists the evaluation metrics themselves.

Among the key performance metrics, latency emerges as the most frequently used metric, appearing in over 50 studies. This underscores the critical importance of minimizing task execution delays, particularly for latency-sensitive applications such as real-time monitoring, autonomous systems, and intelligent IoT services. Energy consumption follows closely, reflecting the widespread need to optimize power usage in resource-constrained devices and fog nodes. Resource utilization is also a prominent metric, used to evaluate how effectively computational and network resources are allocated across the distributed fog infrastructure. In addition, task success rate, which measures the percentage of offloaded tasks completed within QoS constraints, serves as an indicator of system reliability and robustness. Throughput, defined as the number of tasks completed per unit time, is commonly used to assess the efficiency of the DRL agent in managing dynamic workloads. Finally, the cost of a system, in terms of computation, communication, or monetary expenditure, is less frequently evaluated but remains an important consideration for large-scale, real-world deployments. In general, this distribution of metrics reflects the multi-objective nature of DRL-based optimization in fog computing, where trade-offs between latency, energy, and system performance must be carefully balanced.

### 4.5. Comparative Analysis of DRL Algorithms

**Figure 11:**
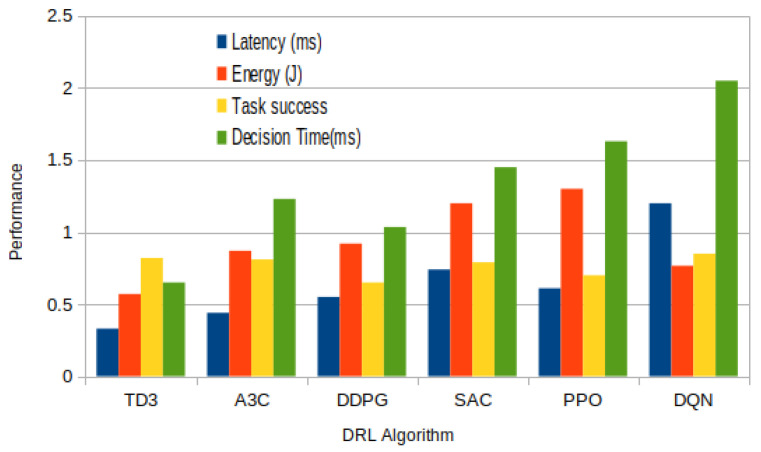
DRL algorithm performance comparison.

Figure 11 presents a comparative analysis of six prominent DRL algorithms—TD3, A3C, DDPG, SAC, PPO, and DQN—based on four performance metrics: latency (ms), energy consumption (J), task success rate, and decision time (ms). This comparison provides valuable insights into the trade-offs and suitability of each algorithm for task offloading in fog computing environments.

Among the algorithms evaluated, TD3 consistently demonstrates the most favorable performance in all four metrics. It achieves the lowest latency and energy consumption, while also maintaining high task success rates and minimal decision time. This suggests that TD3 is well suited for real-time, resource-constrained fog systems, offering both efficiency and responsiveness. In contrast, DQN, despite being the most frequently used algorithm in the literature, performs the worst in terms of latency and decision time, with values peaking above all other methods. While DQN still exhibits a decent task success rate, its high latency and computational overhead indicate that it may not be optimal for highly dynamic or time-sensitive fog scenarios.

PPO and SAC show moderate performance, with PPO slightly outperforming SAC in energy efficiency and task success, though both suffer from relatively high decision times. These algorithms may offer more stable learning due to their policy optimization mechanisms but are potentially less efficient during real-time inference. DDPG and A3C fall in the mid-range across all metrics. DDPG provides a balanced trade-off between latency and task success, while A3C exhibits slightly higher energy consumption. These results highlight their viability for fog systems that require asynchronous training or continuous action spaces, though not necessarily under tight time constraints.

Overall, the analysis underscores TD3’s superiority in balancing all key performance indicators, low latency, energy efficiency, fast decision-making, and high task success, making it particularly advantageous for real-time, distributed fog computing applications. Meanwhile, DQN’s high computational cost, despite its popularity, reveals a gap between theoretical preference and practical suitability.

### 4.6. Single-Agent vs. Multi-Agent Performance

**Figure 12:**
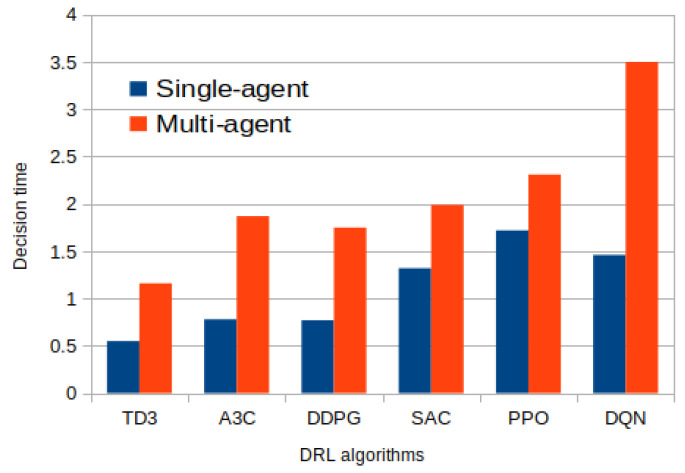
Decision time: single-agent vs. multi-agent DRL.

Figure 12 illustrates a comparative analysis of decision time between single-agent and multi-agent deep reinforcement learning (DRL) algorithms within the context of task offloading in fog computing environments. The results clearly indicate that single-agent DRL algorithms generally exhibit a lower decision time compared to their multi-agent counterparts. This performance difference can be attributed to the simpler decision-making process in single-agent systems, where only one agent interacts with the environment and makes task offloading decisions without the need for coordination or communication with other agents.

Among all evaluated algorithms, TD3 consistently outperforms the others in both single-agent and multi-agent settings. TD3 achieves the lowest decision latency, making it highly suitable for real-time applications in fog computing, where rapid responsiveness is critical to meet strict QoS requirements. Its efficient decision-making can be credited to its core algorithmic improvements, such as delayed policy updates, clipped double Q-learning, and target policy smoothing, which enhance training stability and inference speed.

On the other end of the spectrum, DQN registers the highest decision time latency in both configurations. This performance drawback highlights the limitations of value-based methods like DQN in dynamic, resource-constrained environments where swift adaptation is essential. The relatively high computational complexity and sequential decision-making structure of DQN likely contribute to its slower performance.

The comparison emphasizes a crucial trade-off: While multi-agent DRL (MADRL) systems are advantageous for distributed learning and scalability in fog networks, they introduce additional communication overhead and coordination complexity, which can negatively impact decision time. Therefore, for time-sensitive applications, single-agent DRL, particularly TD3, offers a more efficient and practical solution, whereas MADRL frameworks may be better suited for scenarios requiring decentralized coordination and long-term global optimization.

### 4.7. Task Offloading Decision Time

The task offloading decision time can also be further analyzed by categorizing the agent’s action space into discrete, continuous, and hybrid types, as summarized in Table 3. This classification provides deeper insight into how the nature of the action space directly influences the complexity and speed of the decision-making process in DRL-based offloading systems.

Discrete action spaces typically involve a limited set of predefined choices, such as selecting whether to offload a task or determining which fog node to send the task to. DRL algorithms operating in discrete spaces (e.g., DQN) are generally easier to implement but may face scalability limitations in complex environments, and they often incur higher decision latency as the number of actions increases. Continuous action spaces, on the other hand, allow for fine-grained control over decisions such as adjusting bandwidth allocation or CPU utilization levels. Algorithms such as DDPG, TD3, and SAC are well suited for continuous spaces, enabling more nuanced and adaptive offloading strategies. These models often demonstrate improved efficiency and faster decision times compared to discrete approaches, especially in dynamic fog environments where resource conditions vary continuously. Hybrid action spaces combine both discrete and continuous elements, allowing agents to perform complex multi-dimensional decisions, for example, choosing a target node (discrete) and allocating a specific amount of CPU resources (continuous). While offering greater flexibility and decision accuracy, hybrid models also introduce higher computational complexity, which can impact decision time unless optimized carefully.

By categorizing action spaces in this manner, researchers and practitioners can better select appropriate DRL models based on application requirements, system constraints, and performance goals—particularly with respect to decision latency in real-time fog computing scenarios.

## 5. Discussion

In this section, we critically analyze and interpret the findings presented in the previous sections, linking them to the overarching objectives of this study. We present the taxonomy of DL-, RL-, and DRL-based task offloading in fog computing. After that, we thoroughly examine how the reviewed approaches, frameworks, and methodologies contribute to advancing DRL-based task offloading and resource allocation in fog computing environments.

### 5.1. Taxonomy of DRL-Based Task Offloading in Fog Computing

Recently, task offloading decisions and resource allocation processes between smart user devices and fog computing environments have been supported by AI/ML. This intelligence-based decision approach is used to help the user device choose an offloading target from the available fog nodes. This improves the task offloading process in terms of enhancing resource allocation and task execution delay from the end device to the fog node. Computational tasks and resources are two fundamental elements to consider in fog-based task offloading approaches. In addition, knowing the nature of the application as delay tolerant and delay sensitive affects the level of decision performance. Currently, the application of AI/ML plays a significant role in optimizing the task offloading decision process while adopting an optimal policy. The integration of DL and RL, called DRL, is used to advance the decision process. In addition, hybrid learning techniques are used to solve offloading decision problems.

**Figure 13:**
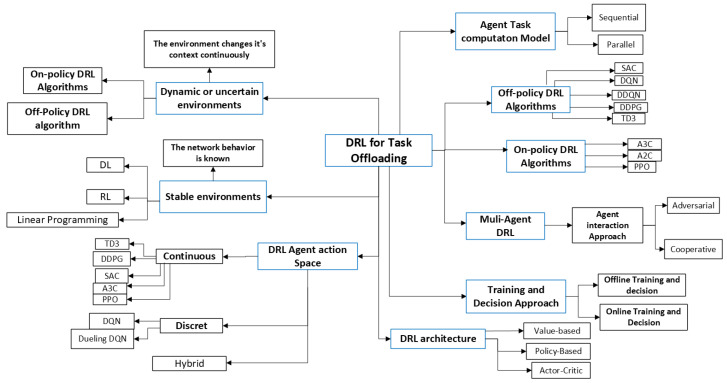
Taxonomy of DL-, RL-, and DRL-based offloading.

Figure 13 depicts the taxonomy of DL-, RL-, and DRL-based task offloading for stable and unstable fog computing environments. This work considers secure offloading as a future exploitable area that has not been covered much.

The key is to explore the knowledge of fog node resources and task requirements for efficient resource allocation. Recently, learning algorithms have been used to extract knowledge from networks. In this regard, Cho and Xiao [54] discussed a learning approach for the relationship between task resource demand and the current workload status of the active fog node in a volatile vehicular computing network. In addition, Qi et al. [55] claimed a knowledge-driven service offloading decision in heterogeneous computing resources using DRL, considering access network, user mobility, and data dependency. Wang et al. [56] presented task offloading in a joint task scheduling and resource allocation problem to design an optimal policy using a deep Q-learning approach for NOMA-based fog computing networks. In the following subsections, the major components of DRL, such as DL, RL, and hybrid learning approaches, are discussed to address the task offloading problem while optimizing multiple objective QoS requirements.

#### 5.1.1. Deep Learning (DL) Approaches

**Figure 14:**
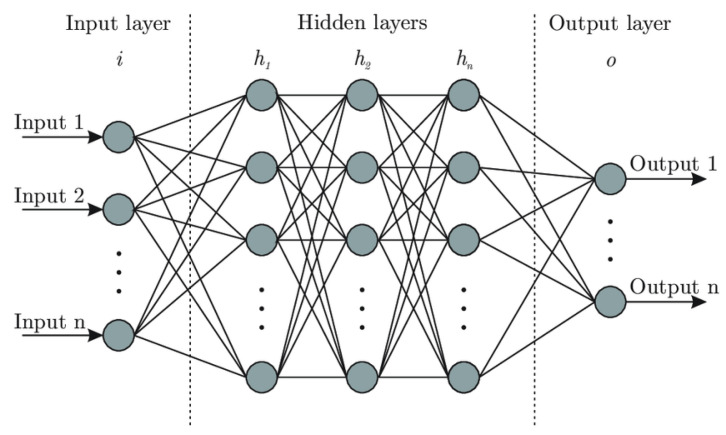
Classical neural network architecture.

DL is a subset of machine learning that uses artificial neural networks (ANNs) with deep architectures (i.e., multiple hidden layers) to automatically learn hierarchical feature representations from data. As shown in Figure 14, a typical DL architecture consists of three main components: an input layer, hidden layers, and an output layer. The input layer maps raw features into the network; the hidden layers process the weighted feature inputs through activation functions to detect complex patterns; and the output layer produces final predictions based on the learning objective. Training is achieved through backpropagation, an optimization algorithm that adjusts the network’s weights by minimizing the difference between predicted and actual outputs.

DL models in fog computing environments, particularly when pre-trained, can significantly enhance network intelligence by supporting efficient task offloading and resource allocation. These models often leverage historical offloading data, such as device behavior, fog node availability, and resource usage patterns, to reduce computational overhead during inference. To improve adaptability in dynamic contexts, lightweight online training can be employed to fine-tune models for specific tasks, enabling situation-aware and real-time decision-making. By capturing complex patterns in the input data, DL facilitates collaborative processing between fog nodes and end-user devices, ultimately optimizing system performance under fluctuating conditions.

Several studies have demonstrated the practical utility of DL in latency-sensitive and mission-critical fog computing applications [49,57,58,59]. Despite these promising developments, the application of DL in highly dynamic fog environments remains limited. Task offloading in such contexts is often stochastic, following the Markov decision process (MDP) framework [60], where task arrivals and system states vary across time and location. Pre-trained DL models, while effective in static settings, often fail to generalize under these dynamic conditions without frequent updates. Maintaining Quality of Service (QoS) thus requires continual model retraining, which can introduce significant computational overhead. Moreover, intermittent fog node availability, particularly in mobile or vehicular networks such as the Internet of Vehicles (IoV), further complicates consistent model deployment and real-time responsiveness.

#### 5.1.2. Reinforcement Learning (RL) Approaches

RL is a subfield of machine learning in which an agent learns to make decisions by continuously interacting with its environment. Through trial and error, the agent receives feedback in the form of rewards or penalties, which guide it in learning an optimal policy, a mapping from environmental states to actions, that maximizes the cumulative reward over time [16]. An RL model is typically defined by six key components: the agent, environment, state, action, reward, and state transition. In the context of fog computing, the environment represents the operational space involving user devices, edge servers, and fog nodes. The state is defined by the contextual information observed from the environment, such as the available computational resources of fog nodes, network bandwidth, latency conditions, and energy levels. The agent—typically a learning algorithm deployed on a user device or controller—perceives this state information and makes decisions about whether or not to offload a computational task.

When the agent takes an action (e.g., offloading to a specific fog node or choosing local execution), it observes the consequences of that action through reward signals. These rewards may be positive (e.g., low latency, efficient resource use) or negative (e.g., task failure, long execution time, high energy consumption). Over time, the agent refines its policy through a balance of exploration (trying new actions to discover their effects) and exploitation (choosing known actions that yield high rewards). The system then transitions to a new state based on the action taken and the environmental dynamics—this is referred to as the state transition.

In RL-based systems, the learning process can be implemented through a variety of training strategies, each offering distinct advantages depending on the complexity of the environment and the problem being addressed. One of the most widely used approaches is the value-based method, such as Q-learning, where the agent learns to estimate the expected utility or “value” of taking a specific action in a given state. This estimation is used to guide the agent toward actions that yield the highest long-term rewards. Alternatively, policy-based methods allow the agent to directly learn and optimize the policy function—a mapping from states to actions—without explicitly computing value functions. These methods are particularly useful in environments with large or continuous action spaces, where value estimation becomes computationally infeasible or less effective. A hybrid of the two, known as the actor–critic method, combines both value estimation and policy optimization to leverage the strengths of each.

RL systems may also be categorized based on whether they rely on an internal model of the environment. Model-based RL approaches involve learning or utilizing a predictive model of the environment’s dynamics to simulate future states and rewards, enabling the agent to plan its actions ahead of time. In contrast, model-free RL does not rely on any explicit modeling of the environment, learning policies or value functions directly from interactions with the environment. While model-free methods are generally more flexible and easier to implement, model-based approaches often require fewer samples and can be more efficient in complex, structured environments.

In the context of fog computing, RL techniques have shown great promise for improving task offloading and resource allocation, particularly in dynamic and unpredictable conditions. The core objective in these scenarios is to train intelligent agents capable of making adaptive, real-time decisions about where tasks should be executed and how computational resources should be managed. RL agents can learn optimal strategies for offloading tasks from user devices to fog nodes or cloud servers by evaluating current system states—such as resource availability, network latency, and energy consumption—and selecting actions that maximize long-term performance. Specifically, RL has been applied in fog environments to minimize end-to-end latency, ensuring that delay-sensitive applications (e.g., real-time health monitoring or autonomous vehicle control) receive timely processing. It also plays a vital role in reducing energy consumption, both on resource-constrained user devices and fog nodes. Furthermore, RL enhances overall system performance by improving task throughput and success rates, and by balancing computational loads across multiple fog nodes, thereby avoiding bottlenecks and increasing the scalability of the fog infrastructure. By continuously learning from experience and adapting to changing network conditions, RL-based task offloading systems contribute to more robust, efficient, and autonomous fog computing architectures.

#### 5.1.3. Hybrid Approaches

Conventional offloading approaches, such as rule-based, linear programming, game theory, heuristics, meta-heuristics, matching theory, and various others, consider between user devices and fog nodes to deal with task offloading decisions and resource allocation problems. In addition, different studies also explore hybrid algorithms, including learning algorithms and conventional approaches, to tackle task offloading decision challenges in the fog computing environment. Some of the representative works are RL with Heuristics [61], Lyapunov guided RL [43], Federated RL [27], DRL with FL [62,63]. Recently, more attention has been given to a combined DL with RL called DRL [9,51], and FL with RL [10,27,63] algorithms are used for offloading problems.

However, the constantly changing formation of the fog and unpredictable updates of services make the environment challenging to converge quickly. To deal with this convergence problem, different learning algorithms and optimization approaches are combined to enhance the offloading decision and resource allocation problem. Lee et al. [61] present RL with heuristic approaches for resource management and resource allocation, respectively. Cao and Cai [64] considered a non-cooperative game approach to deal with communication and computation costs and machine learning to support distributed offloading decisions under dynamic conditions. Most of these approaches focus on global maxima and the immediate performance of the network. However, incorporating collective local and global knowledge can enhance the decision-making process. In conclusion, the application of the DRL algorithm for solving task offloading problems in such stochastic fog environments where the action space continuously grows becomes more applicable.

### 5.2. DRL-Based Task Offloading Architecture in Fog Computing

**Figure 15:**
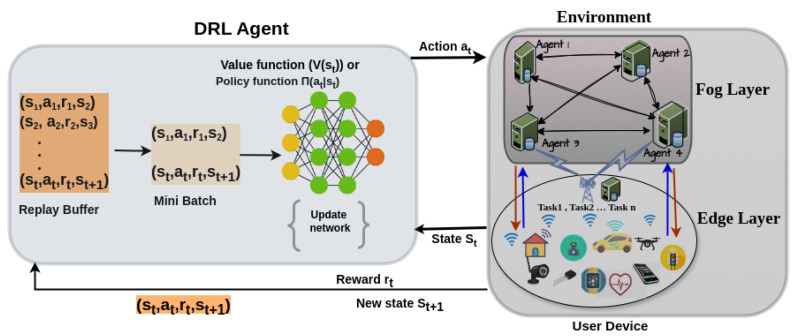
DRL-based offloading architecture.

DRL has become a promising approach for managing task offloading in dynamic and heterogeneous fog computing environments. By integrating the perceptual strengths of DL with the adaptive decision-making of RL, DRL enables agents to learn optimal offloading strategies in real time, even under uncertainty. However, applying DRL in fog computing introduces challenges not encountered in standard DRL applications such as games or robotics, which assume centralized and fully observable environments. Conversely, fog environments are partially observable, distributed, and resource-limited. Each DRL agent is usually deployed at the fog node, and makes a decision based on limited local information while adapting to dynamic workloads, node mobility, and resource heterogeneity. This necessitates decentralized multi-agent DRL (MADRL) architectures, where agents work independently but may cooperate to raise general system performance. The action space and reward structures in fog systems are more complex, mostly encompassing multi-objective targets like energy usage, minimizing latency, and operational cost. Furthermore, due to limited computation and communication resources, DRL models in the fog network have to be lightweight and scalable, leveraging techniques such as online learning or federated updates. Figure 15 illustrates the interaction between DRL agents and the fog environment, where agents continuously monitor task and resource statuses to make informed offloading decisions.

In DRL-based task offloading frameworks, intelligent agents are typically deployed at the fog node level to observe dynamic network states and task characteristics such as workload size, task deadlines, available bandwidth, and energy levels. Based on these observations, the agent makes real-time decisions about whether to execute tasks locally or offload them to neighboring fog nodes. This decision-making process is guided by RL principles, where the agent learns an optimal policy by interacting with the environment and maximizing cumulative rewards over time. As demonstrated in the works of Seid et al. [34] and Xu et al. [35], DRL agents adaptively learn offloading strategies that optimize key performance metrics such as latency, energy consumption, and resource utilization.

#### 5.2.1. Architectural Elements of DRL

In the MDP-based architecture, the task offloading and resource allocation problem in fog computing is formulated as a Markov decision process (MDP). This framework provides a robust foundation for modeling sequential decision-making in dynamic, uncertain, and partially observable environments. Within the context of fog computing, MDP addresses two key challenges: (1) managing state transitions that reflect the stochastic nature of resource availability, and (2) supporting the continuous training and adaptation of reinforcement learning agents under fluctuating system conditions. The MDP model is characterized by essential components such as transition probabilities (*P*), reward functions (*r*), the initial state of each fog node $\left(f_{i}\right)$, and the next state $\left(s^{\prime }\right)$ reached after executing a given action. This formulation enables DRL agents to iteratively interact with their environment, learn from observed outcomes, and gradually develop optimal policies for intelligent task offloading and efficient resource utilization.

**Figure 16:**
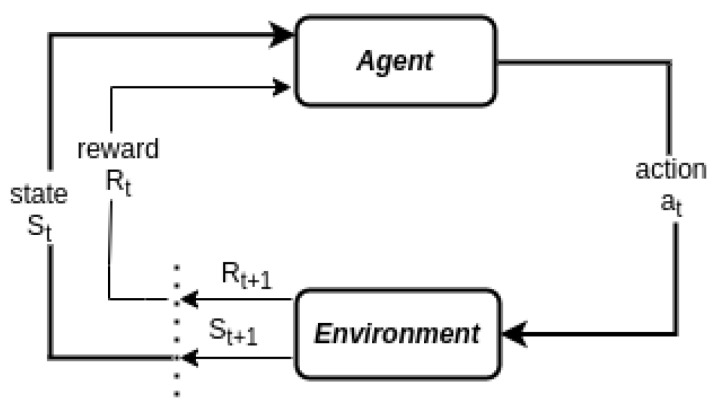
DRL modeling in MDP architecture.

Figure 16 models the learning dynamics formally framed with the MDP tuples (S,A,P,R) where

- *S* is a set of states, where $s_{t}\in S$ is the state in step t.
- A is a set of actions. $a_{t}\in A$ is the action in step *t*.
- $P(s^{\prime }|s,a)$ is the state transition function indicating the probability that state $s^{\prime }$ happens after action *a* is taken in state *s*.
- $R(s,a,s^{\prime })$ is the reward when action *a* is taken in state *s*, and the next state is $s^{\prime }$.

**State:** The state represents the current context or situation of the environment as perceived by the agent. It includes all relevant information needed to make a decision. In fog computing, a state may include variables such as CPU usage, bandwidth availability, energy level, task queue length, and network latency at a fog node.

**DRL Agent:** The agent is the decision-making entity in a DRL system that interacts with the environment. It observes the current state, selects actions based on a learned policy, and updates its strategy over time to maximize long-term cumulative rewards. In fog computing, the agent is typically deployed at the fog node, where it learns when and where to offload tasks or allocate resources. In multi-agent environments, the agent interaction models are described as cooperative, adversarial, mixed (cooperative and competitive), hierarchical, learning-based and communication-based [24,65].

**Action:** In this DRL-based task offloading in fog, the action is defined as executing the task locally and/or offload it to fog nodes. The action is actual, meaning that the selection of fog nodes and allocation of resources is performed according to the agent’s policy within the fog environments. The agents start the action by learning the network from their interactions based on resource status and task QoS requirements. Then, the decision is made either to execute locally or offload to fog nodes. In the second stage, if the user device decides to offload its executable task to the nearest local fog node, the task’s QoS requirements are attached to the task as information. After that, the autonomous fog node decides to execute locally or offload to other fog nodes. When the decision is to offload, the fog node with the task is looking for resources such as bandwidth, computing power, and memory for processing the offloaded task [66]. This exhaustive search of resources causes the search space to grow exponentially and increases run-time processing when task sizes increase in distributed fog computing. In support of this, Baek and Kaddoum [42] used Deep Recurrent Q-network (DRQN) and Liu et al. [46] Deep Q-learning (DQL) for task offloading problems for computation-intensive tasks. They claim that applying continuous updating raises a performance issue for task offloading; it mainly focuses on immediate performance, leading to performance degradation in the long run. Likewise, the stochastic nature of fog computing environments due to the mobility of fog nodes creates uncertainty of connectivity between nodes and makes it difficult to manage the state transitions of nodes. This highlights how the MDP can effectively model the dynamic nature of the network and addresses the challenges of task offloading.

**Reward function:** In DRL, the reward can be positive or negative depending on the agents’ decisions. The goal is to maximize the positive reward by minimizing task completion time and improving resource utilization during the execution of offloaded task. The negative reward is interpreted as the incurring of the maximum cost of resources, such as energy consumption and latency. The agent uses the value function and the policy function to calculate the reward from the agent’s present observation and transition probability distribution of the proposed action. The goal of the agent is to maximize the reward using the local maximum of the policy $\pi^{*}\left(s\right)$ in each state of iteration.

However, in this MDP-based learning process, the agent does not know the direction in which to proceed while gathering the maximum reward. This is where the Bellman Equation (1) applies in RL learning to enable the agent with the memory from the transition in the MDP process.

(1) $$Q\left(s^{\prime },a\right)\leftarrow r+\gamma \cdot \max_{a^{\prime }}Q\left(s^{\prime },a^{\prime }\right)$$

Therefore, Equation (1) essentially states that the optimal policy for a given state (s) is the action that maximizes the sum of the immediate reward and the discounted value of the next state, considering all possible next states $\left(s^{\prime }\right)$ and their associated transition probabilities (p). By applying Equation (1) recursively for each state in the environment, we can obtain the optimal policy for the entire environment. The reward function presented by Liu et al. [46] uses iterative computation of the optimal value function, which represents the maximum expected cumulative reward that an agent can receive from a given state *s* under an optimal policy $\pi^{*}\left(s\right)$. So, it is used to draw the value iteration $\left(V_{i}\right)$ in each state. It starts by initializing the value function $V^{\left(0\right)}\left(s\right)$ to 0 for all states *s*. Then, for each iteration *i*, the algorithm updates the value function $V_{\left(i+1\right)}\left(s\right)$ for each state *s* by taking the maximum expected cumulative reward over all possible actions that can be taken from that state *s*. In the reward function, a hyperparameter α and γ are used to measure how far the agent reaches its goal and to govern the algorithm’s pace for updating the value parameters, respectively.

#### 5.2.2. Single-Agent vs. Multi-Agent DRL

Fog computing is inherently distributed, relying on decentralized management for low-latency and scalable service delivery. In such architectures, users can execute their applications on one or more fog nodes. To support intelligent offloading and efficient resource allocation, AI/ML methods are increasingly adopted. However, in these single-agent models, task and resource management can hinder performance in decentralized fog environments where nodes operate autonomously. Multi-agent learning (MAL) has emerged as a robust alternative, enabling fog nodes to coordinate their decisions. This cooperation helps reduce computational delays and enhances the overall utilization of resources across the system. Here, the challenge is how to coordinate the agent policy and inter-agent communications. Two MAL settings are considered in RL-based task processing: The first is the cooperative approach, where the fog nodes (DRL agents) collaborate to achieve common objectives; agents share information to achieve the best result for the team. The other approach is the adversarial, in which agents compete to achieve their individual goals. Recently, DRL enabled MAL to be applied to various problem domains. The MADRL used in MEC enabled the Industrial Internet of Things (IIoT) for multi-channel access and task offloading problems [67]. Lu et al. [68] designed a multi-agent DRL approach for independent learning agents to solve stochastic end-user massive requirements change and computational resources. Chen et al. [40] introduced cooperative MADRL for multi-device multi-cloud dynamic network settings for real-time computing requirements in varied wireless channels in a centralized training and distributed execution strategy.

### 5.3. Task Offloading Architecture

The architectural scope of task offloading in fog computing defines the structural arrangement and interaction between user devices $U_{n}$, fog nodes $F_{m}$, and cloud computing (CC) infrastructure. These architectures determine where and how computational tasks are offloaded and processed, which has direct implications for latency, energy efficiency, bandwidth utilization, and QoS. They also influence how learning-based offloading decisions are designed, specifically, how models explore and exploit task-resource coordination in distributed environments. In the user device–to-fog setup, tasks are offloaded directly from IoT devices to nearby fog nodes, which provide low-latency processing capabilities closer to the edge. In contrast, the user device–to-fog–to-cloud architecture introduces a hierarchical processing framework, where tasks are first offloaded to fog nodes, and if resource constraints are encountered, they are further offloaded to the cloud. The user device–to-cloud model bypass intermediate fog layers, sending tasks directly to the cloud, which may result in higher latency but greater computational power. Lastly, the fog–to-cloud architecture supports inter-layer communication between fog and cloud for load balancing, resource sharing, and overflow handling.

**Figure 17:**
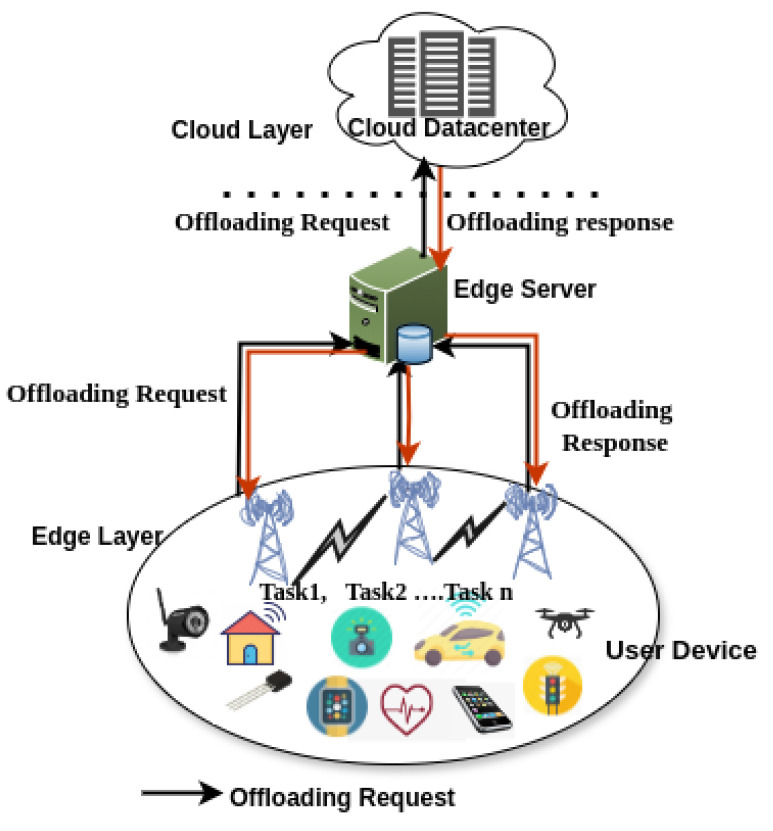
Task offloading architecture where multiple user devices offload their tasks to a single central fog server.

**Figure 18:**
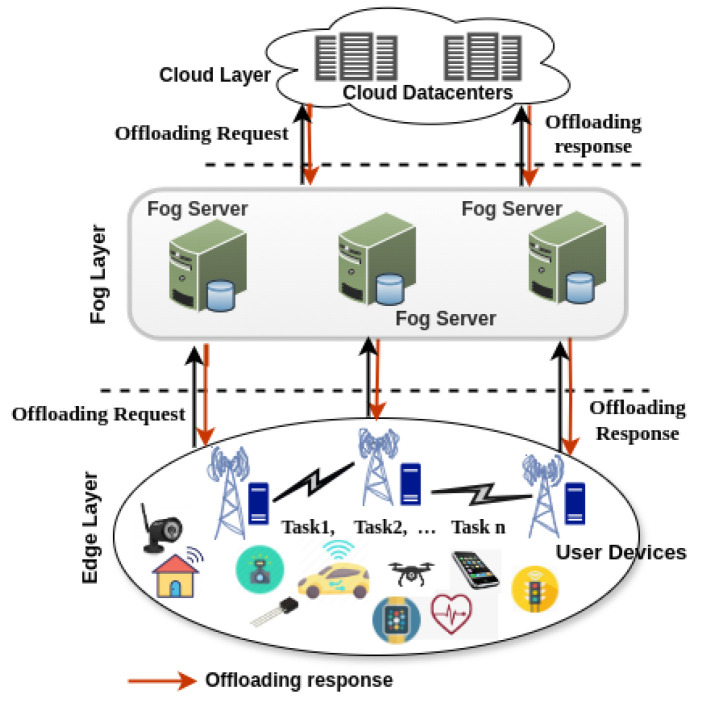
Task offloading architecture where multiple user devices offload their tasks to multiple fog nodes, but there is no cooperation between fog nodes.

**Figure 19:**
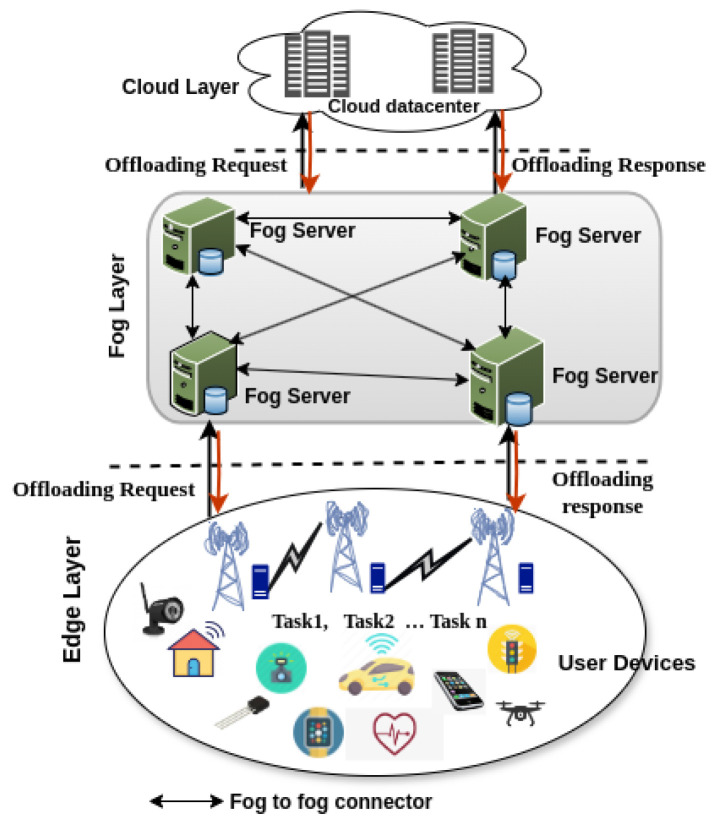
Task offloading architecture where multiple user devices offload their tasks to multiple cooperating fog nodes.

The current task offloading scenarios in fog computing are illustrated through the three architectures shown in Figure 17 (many-to-one), Figure 18 (many-to-many without fog-to-fog), and Figure 19 (many-to-many with fog-to-fog), where user devices offload computational tasks to fog nodes for processing.

Figure 17 illustrates a many-to-one offloading approach, where multiple end-user devices offload their tasks to a single edge server, as presented by Hortelano et al. [1]. This configuration primarily represents an edge computing scenario, as it involves only one computing node positioned above the user devices. In contrast, a true fog architecture should involve multiple interconnected computing nodes within the fog layer to support distributed processing. Figure 18, adapted from Zabihi et al. [69], depicts a many-to-many task offloading model, where multiple user devices offload tasks to multiple fog servers using various communication protocols. However, this architecture does not address inter-fog node communication, which is essential for dynamic task migration and resource balancing across the network. Similarly, Figure 19, based on the work of Tran-Dang et al. [12], presents another many-to-many offloading architecture that introduces the concept of communication between fog nodes in the network. However, the mechanisms of fog-to-fog communication, particularly how fog nodes interact and collaborate, are not explicitly detailed.

Overall, the architectural scope plays a critical role in shaping the effectiveness of learning-based task offloading frameworks. A robust decision-making model must align with the operational characteristics of the deployed architecture, whether flat (e.g., device–fog) or hierarchical (e.g., device–fog–cloud), to ensure optimal performance in distributed fog computing environments. Based on this comparative analysis, this study recommends the extended many-to-many task offloading architecture (Figure 19) as the most suitable model for applying DRL in fog computing environments. Unlike previous models, it incorporates the critical feature of inter-fog node communication, which is essential for collaborative decision-making, dynamic task migration, and efficient resource balancing. This architecture best aligns with the decentralized and latency-sensitive nature of fog computing and offers the structural flexibility needed for DRL-based task offloading in dynamic IoT systems [38].

### 5.4. Agent Controller Strategies

The coordination of agent communication between the task-owned node and the task executor node is discussed in the literature as a centralized, distributed, and federated approach. In the centralized model, a central controller is responsible for making all task offloading decisions. The central controller gathers information from various fog nodes to assign tasks and monitor execution across the system. Both model training and decision-making processes occur at the central server. While this architecture simplifies coordination, it poses significant scalability challenges, including the risk of a single point of failure. Moreover, it is generally unsuitable for dynamic, latency-sensitive applications or scenarios that require real-time, on-the-fly processing of raw data. The model is most effective when computational resources are managed by a single entity or institution [70].

The distributed architecture typically follows one of two approaches. In the first, task computation is delegated to multiple fog nodes, while offloading decisions are still managed centrally. In the second, both task computation and offloading decisions are handled in a fully distributed manner. For the latter approach, distributed learning techniques are employed to support the offloading process by sharing communication and execution information across nodes. Each fog node is responsible for managing the resource collaboration and executing tasks, thereby enabling decentralized control of the offloading process [10,71,72]. However, this model introduces certain challenges. Offloading private data to unknown or untrusted fog nodes raises security and privacy concerns. Additionally, frequent task transmissions can increase network traffic, leading to potential performance degradation across the system.

Traditional centralized and distributed learning approaches typically involve frequent offloading of locally generated data from user devices to edge, fog, or cloud servers for training and decision-making. Wahab et al. [73] introduced federated learning as an alternative offloading strategy to address critical issues such as network traffic, data privacy, and security. In federated architectures, model training is performed locally on user devices or within a heterogeneous network environment, avoiding the need to transmit raw data to fog nodes. Instead, only the trained model or its updates are shared with distributed nodes, where decision-making can also occur. This approach significantly enhances data privacy and security while reducing communication overhead, making it well-suited for latency-sensitive and privacy-critical applications.

### 5.5. Coordination Mechanisms

Task offloading architectures are typically classified according to the coordination mechanisms among nodes that provide computational resources for executing tasks. These architectures fall into three main topological categories: (i) static (fixed) topology, (ii) dynamic topology characterized by node mobility, and (iii) hybrid topology, which combines features of both static and dynamic configurations. This section examines each of these architectural models, discussing their practical application scenarios, the unique challenges they present, and the solutions proposed in the existing literature.

**Static Model**: In this architectural approach, both the fog nodes and the client user devices’ status and the placement remain unchanged over time. Here, the architecture follows a controlled approach since the task offloading node and receiving fog nodes are known. The representative application scenarios mentioned, such as manufacturing industries [74], involve fog nodes and IoT devices such as cameras and sensors that are deployed in fixed locations. Furthermore, smart traffic monitoring scenarios for stationary traffic nodes are also considered an example of fixed topology, since both the cameras and traffic light sensors are installed in a constant location [75]. Smart city utilities such as public safety, optimized service delivery, and others [76] can be designed with this type of architecture. Resource management in this architectural model exhibits lower complexity compared to dynamic and hybrid models [20]; however, potential concerns relate to underutilized resources and inflexibility in allocating them for collaborative tasks involving other devices.

**Dynamic Model**: In this architectural model, the topology designed for both the condition of servers and clients is constantly changing their status [77]. This dynamism reflects both the resource status of the participating nodes and their mobility in joining or leaving the network, such as in vehicular fog computing. This means that a node’s status is unpredictable in terms of its workload, location, and resource availability. This mobility-aware fog computing network setting considers key features such as dynamic resource allocation, node mobility predictions, managing seamless offloading tasks and data, context-aware computing, and others [78]. Particularly, it ensures service continuity and resource utilization between dynamic fog nodes when devices move from one coverage area to another by leveraging location and contextual information for optimizing task processing and decision-making at the respective nodes. Tan et al. [79] examined task offloading in dynamic fog computing networks with non-stationary topologies and variable computing resources, comparing stationary and non-stationary contexts. While their model assumes constant unit-task offloading delay under static or slow-changing conditions, real-world node mobility—characterized by unpredictable speed, distance, and duration within coverage—requires intelligent handling. In multi-hop ad hoc fog environments, such mobility can significantly impact application performance. Similar concepts have been applied in UAV-based fog computing for the Internet of Medical Things (IoMT) in rural, resource-limited areas [80] and in vehicular fog computing [61], where both task-requesting and task-executing nodes are in motion.

**Hybrid Model**: The hybrid model is where the network context has a mix of dynamic and static models. In conventional hybrid fog architecture, the fog nodes typically consist of fixed devices, whereas the user devices are mobile [81]. However, in this mixed network setting, dynamic and static settings are introduced, and the architecture becomes even more dynamic and requires additional considerations. This category considers the fog node as having a constant location as static, and clients’ status is mobile or vice versa according to priority settings [82]. The application scenarios for this model are represented as traffic monitoring and surveillance [83], environmental monitoring [84], and UAV-based service provision mechanisms in a rural area presented in [85], where there is no stable network connection. The UAV acts as a server to provide an access point for the local end devices. It can also be applicable to provide communication services in emergency response in a disaster context. Hazra et al. [81] explain the use case of a health monitoring environment where fog nodes are fixed, but patient status monitoring IoT devices are in mobility modes. The opportunities in the mixed architectural model are the separation of layers, simplifying resource management, improved security, and data privacy through local processing in each context. The challenge in this fixed architecture is potentially higher upfront costs compared with other approaches.

In dynamic and hybrid architectural models, managing the complexity of mobile fog nodes requires advanced control mechanisms. These nodes often have limited battery life, making efficient task processing and communication protocols essential for optimization. Their unstable nature can lead to task failures, disrupting dependent tasks offloaded to other nodes. Ensuring secure communication and data privacy in such dynamic environments is also a key challenge. To address these issues, learning-based mechanisms that predict node mobility patterns and network characteristics—such as long-term connectivity and stable resource availability—are increasingly necessary. Recent research proposes distributed, decentralized control strategies to adapt to changing network conditions, with emerging focus areas including self-organizing networks and AI-driven resource management.

An important aspect in these models is awareness of fog node and resource status for effective task offloading. Pu et al. [86] introduced a device-to-device (D2D) communication approach to enable collaboration between edge devices, supported by an incentive scheme similar to peer-to-peer networks. Similarly, Al-Khafajiy [87] proposed collaborative strategies between fog nodes for shared workload processing. Through D2D communication, IoT devices can exchange status information with nearby devices to identify idle resources, enabling adjacent devices and fog nodes to coordinate via a wireless access point. In fixed-to-fixed architectures, device routing tables advertise and update resource status for all connected nodes. In both cases, the collected knowledge supports informed offloading decisions. Future work should explore DRL-based self-organizing network settings for smart user devices and fog computing environments that integrate both fixed and dynamic architectures.

### 5.6. DRL Algorithms

To provide contextual understanding, RL is broadly classified into model-based and model-free approaches. Model-based RL leverages knowledge of the environment’s dynamics, typically represented by an MDP with transition probabilities $P(s^{\prime }|s,a)$, in terms of policy iteration $\pi_{\theta }\left(s\right)$ and value iteration V(s) to guide decision-making in stochastic fog computing environments. In contrast, model-free RL does not assume prior knowledge of the environment’s dynamics. Instead, it learns optimal policies through interaction, using methods such as policy gradient optimization, off-policy algorithms like Q-learning Q(s,a), and on-policy algorithms including Temporal Difference (TD) learning and SARSA. These approaches are widely used for task offloading and resource management in dynamic fog environments.

DRL extends both paradigms by integrating deep neural networks to approximate policies or value functions, enabling scalability to high-dimensional state and action spaces. Examples include Deep Q-Networks (DQN), which are model-free, and Actor–Critic (AC) methods, which combine policy and value learning. Some DRL frameworks also incorporate model-based components for planning and prediction. This hybridization improves adaptability and efficiency in complex, dynamic systems such as fog computing.

Several notable DRL algorithms have been applied to address the task offloading problem in fog computing, as summarized in Table 4. These algorithms can be categorized into Value-Based (VB), Policy-Based (PB), and Actor–Critic (AC) methods. VB algorithms focus on estimating the value of being in a particular state or performing a specific action. A prominent example is the Deep Q-Network (DQN), which integrates Q-learning with deep neural networks. It incorporates mechanisms such as experience replay and target networks, making it well suited for environments with discrete action spaces.

PB methods, on the other hand, learn a policy function directly by optimizing the probability distribution over actions. An example is the Asynchronous Advantage Actor-Critic (A3C), a multi-threaded version of A2C that enables faster and more stable training through parallelism. In contrast, AC approaches combine elements of both value-based and policy-based strategies. The Proximal Policy Optimization (PPO) algorithm is widely used due to its simplicity and robust performance, employing a clipped surrogate objective to enhance training stability. The Deep Deterministic Policy Gradient (DDPG) algorithm, designed for continuous action spaces, uses deterministic policies and an actor–critic framework. Twin Delayed DDPG (TD3) further improves upon DDPG by introducing twin critics and delayed policy updates to reduce overestimation bias and enhance stability.

### 5.7. Key Input Parameters and Learning Mechanisms in DRL

In DRL-based task offloading, input features, also referred to as state variables, capture the current system state that the agent observes and uses to make decisions. These features form the basis of the agent’s decision-making process, enabling it to adapt to changing conditions in dynamic fog computing environments. In practical implementations, DRL models can incorporate a diverse set of input features, such as CPU capacity, memory usage, channel conditions, energy levels, and application deadlines. By leveraging both historical data and real-time measurements, offloading decisions become more adaptive and context-aware, allowing the system to optimize resource utilization and maintain high Quality of Service (QoS) under varying conditions.

A key learning mechanism in DRL is the replay buffer, which stores the agent’s past experiences, including offloading decisions, system states, and associated rewards. During training, these stored experiences are randomly sampled and reused, enhancing learning stability, improving sample efficiency, and preventing the model from overfitting to recent interactions. This mechanism also enables better generalization to unseen scenarios, which is particularly valuable in non-stationary environments—such as fog networks—where resource availability and task patterns change frequently.

By integrating rich state information with robust learning mechanisms like the replay buffer, DRL-based offloading strategies can achieve both stability and adaptability, ensuring more reliable decision-making in complex, real-world deployments.

### 5.8. Task Execution Models

Due to the resource limitations of user devices, a key challenge lies in deciding the optimal execution location for a task, balancing trade-offs among competing QoS objectives in practical deployments. The literature identifies two primary application scenarios in this context. A task may be offloaded or processed locally based on real-time system constraints and application demands, as discussed below.

#### 5.8.1. Local Execution Model

Computing tasks locally on an end-user device cancels overheads such as communication costs. However, the energy consumption and computing power constraints on local user devices while running the application locally incur reliability and performance problems [94,95]. The decision criteria to be considered for local task execution can be computation time, energy consumption, device capabilities, and data privacy. For example, the low battery lifetime and limited computing power in the end-user device affect performance while achieving QoS objectives. In the literature, researchers consider both local user device resources and offloading some tasks to the fog node to utilize the local resources while boosting performance. The task is divided into numerous sub-tasks, which are then assigned to the local device for local execution. The remaining tasks are then offloaded to fog nodes. Local execution overhead is expressed in terms of execution time (required CPU cycles and computing capability) and local energy consumption.

Extracting both the historical and current task execution status of devices, along with their available resources, enables more informed offloading decisions. Building on this concept, Zhu et al. [59] proposed a computation offloading mechanism aimed at minimizing task completion time and energy consumption within the local fog environment. As reported in previous studies, local execution time and energy consumption overhead are measured by considering the utilization of the CPU cycle and computing capabilities of user devices. In this regard, Wang et al. [56] presented a mathematical model to represent the local energy consumption of IoT devices, which is determined by the number of required CPU cycles and the energy consumed per cycle. As a result, the cost of local execution overhead of end devices can be obtained from the summation of the weight of local execution overhead in terms of the local weight of energy consumption and the weight of task execution delay. Assume that $D_{i}^{l}$, $f_{i}^{l}$, and $C_{i}$ denote the local computation delay to compute task $\beta_{i}$, computing capabilities of the task node *i*, and the number of CPUs, respectively. So, $D_{i}^{l}=\frac{C_{i}}{f_{i}^{l}}+Q_{i}^{t}$, where $C_{i}$ depends on the size of the task $T_{i}$ and considers the queue time for each task $Q_{i}^{\beta }$. In addition, the energy consumption per CPU cycle $\zeta_{i}$ and the energy consumption for computing tasks $E_{i}^{l}$ are considered to calculate the energy cost as $E_{i}^{l}=\zeta_{i}C_{i}$. The overall cost of the local task execution $\Omega_{i}^{l}$ is obtained from the weight of the delay in the execution of the local task plus the weight of the energy consumption cost for that particular task.

(2) $$\Omega_{i}^{l}=W_{i}^{t}D_{i}^{l}+W_{i}^{e}E_{i}^{l}$$

$W_{i}^{t}$ and $W_{i}^{e}$ are the weights of local task execution delay and energy consumption, respectively. Equation (2) allows us to compare the cost of task execution with offloading approaches. Wang et al. [56] propose a learning-based partial offloading approach. That means parallel use of local computing resources to avoid communication and offloading costs, whereas the offloading model is used to benefit from the rich fog computing resources.

Note that, in multi-agent learning, task execution can follow either a sequential or parallel approach, depending on the learning algorithm and the interaction pattern between agents. Sequential execution is suited to environments where agents learn independently from their own experiences without direct interaction, or in hierarchical settings where higher-level agents make decisions that guide the learning of lower-level agents in a step-by-step manner. This method is often preferred when computing resources are limited. In contrast, parallel execution enables agents to learn simultaneously while interacting and exchanging information, allowing them to influence each other’s learning and actions in real time.

#### 5.8.2. Task Offloading Model

The application of smart user devices grows in a wider perspective, despite that their limited resources pose performance problems. Due to resource constraints on the end user devices, delay-sensitive applications are unable to execute tasks while fulfilling their QoS requirements, such as energy consumption and task computation deadline [60,96]. To handle this problem, offloading computational tasks to resource-rich fog nodes is a promising approach to meet QoS requirements for delay-sensitive applications. This by far reduces local execution overhead while considering communication and transmission costs. If the decision is to offload the task to the fog nodes, the major question becomes determining where to offload a task among the distributed fog nodes. The decision criteria to offload the task to the fog node need careful consideration. In this case, the decision metrics can be fog node network resource, communication cost, computation cost, task latency requirements, fog node availability, resource availability, performance requirements, and task complexity. Wang et al. [56] also discuss the model of mathematical equations to calculate and analyze each computational aspect concerning the offloading cost. Starting from task transmission delay to each node *i* can be computed as $D_{i,t}^{o}=\frac{T_{i}}{r_{i}}$, and computation delay as $D_{i,p}^{o}=\frac{C_{i}}{f_{i}}$, where $T_{i}$, *p*, and $r_{i}$ are task size, transmit power, and achievable rate for the ith task node, respectively. With that, the energy cost can be given as follows in Equation (3):

(3) $$E_{i,t}^{\left(o\right)}=p_{i}D_{i,t}^{\left(o\right)}$$

where $p_{i}$ is the idle time after task offload to the ith fog node. The total cost of offloading $\Omega_{i}^{o}$ the task node *i* can be calculated as follows using Equation (4):

(4) $$\Omega_{i}^{o}=w_{i}^{t}\left(D_{i,t}^{o}+D_{i,p}^{o}\right)+w_{i}^{e}\left(\frac{p_{i}T_{i}}{r_{i}}+\frac{p_{i}C_{i}}{f_{i}}\right)$$

Energy consumption is measured at the idle time $p_{i}$ as $E_{i,p}^{\left(o\right)}=\frac{p_{i}C_{i}}{f_{i}}$ where $f_{i}$ is the assigned computational resources, and this should not exceed the total fog node resources. $\sum_{i=1}^{K}\delta_{i}f_{i}\leq F$, Here δ is the offloading decision parameter, where δ=0 indicates that the task is executed at the local node and δ=1 indicates that the task is offloaded to the fog node. Subsequently, the total cost of task nodes is expressed in Equation (5), taking into account both the delay and the cost of energy consumption outlined as follows.

(5) $$\Omega_{all}=\sum_{i=1}^{I}\left(\left(1-\delta_{i}\right)\Omega_{i}^{l}+\delta_{i}\Omega_{i}^{o}\right)$$

However, a central challenge lies in how task offloading decisions are coordinated between user devices and fog nodes. Effective task offloading and execution must be managed with respect to available computing resources and QoS objectives. In the literature, three primary offloading decision approaches have been identified: partial offloading [97], full offloading [98], and preference-based offloading [99].

Generally, in the process of offloading decisions, the user device needs to know information about the neighboring task-receiving fog nodes. In this context, Al-Khafajiy et al. [87] discuss the request offloading method with a collaboration strategy among fog nodes to balance the fog computation overload. Mostly, the offloading decision problem is associated with communication delay, task queuing delay, and execution delay [88], plus the types of application.

#### 5.8.3. Illustration of Task Offloading Decision

**Figure 20:**
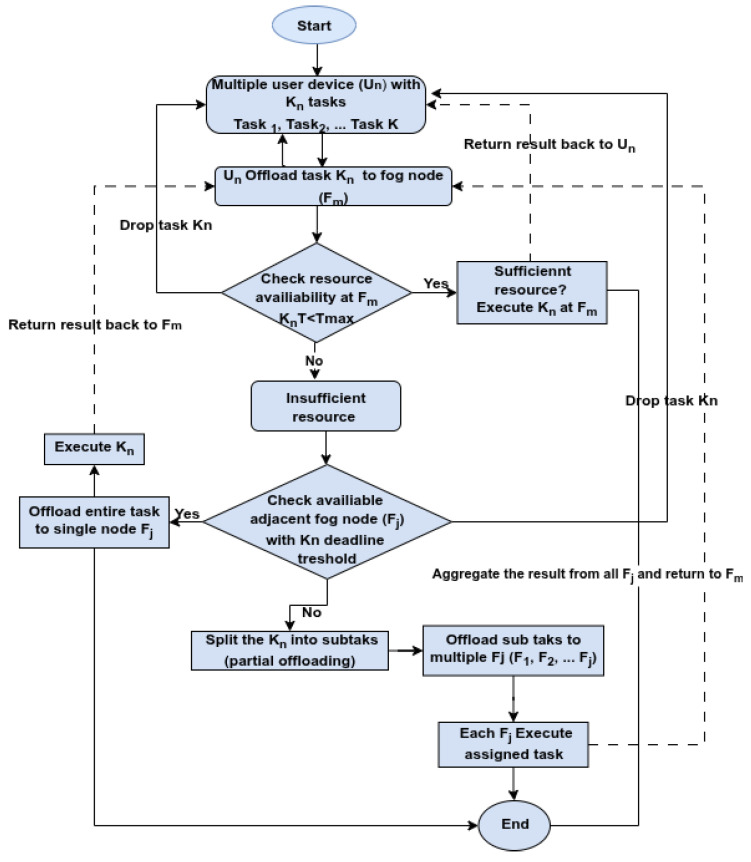
Task offloading flow chart.

Figure 20 presents the task offloading flowchart for a fog computing environment involving multiple user devices and fog nodes. The process begins with multiple user devices $U_{n}$, each generating a set of tasks $K_{1},K_{2},\ldots ,K_{n}$. Each task $K_{n}$ is offloaded from the user device $U_{n}$ to a designated fog node $F_{m}$ for execution. Upon receiving a task, the fog node $F_{m}$ first checks whether it has sufficient resources to execute $K_{n}$ within its maximum allowable deadline $T_{\max}$. If both conditions are satisfied, the task is executed at $F_{m}$, and the result is returned to the originating user device $U_{n}$.

If $F_{m}$ lacks sufficient resources or cannot meet the deadline constraint, the system evaluates alternative execution strategies. First, it checks whether an adjacent fog node $F_{j}$ is available and capable of executing the entire task $K_{n}$ within the deadline. If such a node exists, the task is fully offloaded to $F_{j}$, and the result is returned to $F_{m}$ and then forwarded to the user device $U_{n}$.

In cases where no single adjacent fog node can meet the requirements, the task $K_{n}$ is split into subtasks (partial offloading), which are then distributed to multiple fog nodes $F_{1},F_{2},\ldots ,F_{p}$ for parallel execution. Each fog node executes its assigned subtask, and the results are aggregated and returned to the original fog node $F_{m}$, which then forwards the final output to the user device $U_{n}$.

If no suitable fog nodes are found and the system cannot meet the deadline or resource constraints through either full or partial offloading, the task $K_{n}$ is dropped. The flowchart also includes feedback loops indicating dynamic task reassessment or fallback to earlier stages based on execution outcomes. This flowchart illustrates a hierarchical and adaptive task offloading strategy that supports direct execution, fallback delegation, and distributed partial offloading, making it well suited for dynamic fog computing environments. Moreover, this structure provides a strong foundation for deep reinforcement learning (DRL)-based task offloading, where agents can learn optimal strategies by observing system states, resource availability, task deadlines, and performance outcomes over time.

From a decision-making perspective, user devices typically begin by selecting the nearest or least-loaded fog node for task offloading. The selected fog node then allocates available resources to execute the task and returns the result to the user device. If the fog node lacks sufficient computational capacity or is overloaded, it forwards the task to the cloud for execution. This multi-tier processing model requires intelligent, adaptive decision-making mechanisms capable of learning from the environment to optimize offloading paths and scheduling. As such, the design of learning algorithms for task offloading and resource allocation must be tightly coupled with the architectural setup. For instance, reinforcement learning agents must be trained with awareness of the hierarchical structure (fog vs. cloud), available bandwidth, node availability, and task urgency. The dynamic interplay between architecture and learning model requires that offloading decisions consider factors such as network latency, fog node capacity, task deadlines, and energy constraints.

#### 5.8.4. Validation Metrics

Validation metrics are performance indicators used to evaluate a model during or after training, assessing how effectively it meets the objectives of the task offloading problem. Common metrics in DRL-based fog computing include the following:

- Accuracy—the proportion of correct offloading decisions compared to an optimal or benchmark policy.
- Latency reduction—the decrease in task execution time achieved through optimal offloading.
- Energy efficiency—the reduction in energy consumption of devices and fog nodes.
- Throughput improvement—the increase in the number of successfully processed tasks within a given time frame.
- Task completion delay—the total time required to execute and return task results.
- Reward convergence—how quickly and stably the DRL model’s reward function reaches an optimal value.

Numerous studies have applied these metrics to evaluate system performance. For example, some have prioritized low latency as the primary objective [45,70,88,100,101]. Others have focused on reliability by minimizing offloading costs or balancing trade-offs between energy consumption and delay to optimize overall system cost [56,59,100,101,102]. Additional works address both delay and reliability to reduce network delay costs and enhance offloading decisions [57,74,92,93]. Finally, some studies emphasize energy efficiency to maintain network reliability [103].

By selecting appropriate validation metrics, researchers can align evaluation strategies with the intended operational goals of the DRL model, ensuring that performance improvements translate into tangible benefits in real-world fog computing environments.

### 5.9. Training and Decision Approach

DRL combines the representational power of DL with the adaptive decision-making capabilities of RL. While both contribute to DRL’s effectiveness, the two paradigms differ substantially in their training processes and decision-making mechanisms. DL is typically suited for scenarios with stable network conditions, where a fixed and pre-defined dataset can be used. In such cases, data are collected, features are extracted and labeled, and the dataset is split into training, validation, and testing subsets. The model is then iteratively trained over multiple epochs to optimize performance. In contrast, RL excels in highly dynamic environments where network conditions change continuously, making fixed datasets impractical. RL agents do not require pre-labeled data; instead, they interact directly with the environment, observe states, take actions, and receive rewards, gradually improving their policies through trial and error. DRL extends this process by incorporating a replay buffer, which stores past experiences and randomly samples them during training [90]. This mechanism improves learning stability, enhances sample efficiency, and enables the agent to adapt effectively to non-stationary conditions, such as in fog computing where resource availability and task patterns frequently change.

A key distinction is that DL-based decision-making remains static after training, whereas RL-based decisions evolve continuously as the agent learns from ongoing interactions. The choice between DL, RL, or a hybrid DRL approach depends on the stability of the operating environment and the level of adaptability required.

In fog computing, the DRL training process involves an iterative interaction with the environment, where the agent collects experiences as *state–action–reward* sequences and updates its policy or value function to improve decision-making over time. Depending on application requirements and system constraints, DRL training can follow different paradigms, as summarized in Table 5. From a deployment perspective, DRL models can be trained and applied using several modes:

- Offline Training (Batch RL): uses a static, pre-collected dataset to train the model before deployment. This allows safe policy learning without affecting live systems but limits adaptability to unseen scenarios.
- Online Training: continuously updates the model during real-time interaction with the environment, enabling adaptive decision-making but potentially increasing computational and communication overhead.
- Centralized Training: aggregates all data at a central server for joint model training, offering strong coordination but raising concerns over scalability and privacy.
- Distributed Training: splits the workload across multiple computing nodes, reducing training time and improving scalability though requiring robust synchronization mechanisms.
- Federated Learning-Based DRL: allows multiple agents or devices to collaboratively train a shared model without exchanging raw data, preserving privacy and reducing communication overhead—especially valuable in heterogeneous and sensitive fog computing environments.
- Testing: conducted under both stable and dynamic conditions to evaluate model performance using metrics such as accuracy, latency, energy efficiency, and reliability, ensuring robustness across diverse scenarios.

**Table 5:** Related works on DRL-based task offloading with respect to QoS objectives and training approaches.

Reference	Objective	Algorithms	Decision	Training					Pros (+) and Cons (−)
				Online	Offline	Central	Distributed	Federated	
[90]	Optimize joint performance and cost	DQN	Online		✓		✓		+ Considering dynamic network − Single edge node with no control model
[102]	Enhance network latency to meet response time	CNN	Online		✓		✓		+ Applying distributed decision − Challenging to update model
[56]	Minimize delay and energy consumption cost	DQL	Online	✓		✓			+ Optimize offloading between fog nodes − Unknown helper node status
[89]	Enhance long learning time	DQN	Online	✓			✓		+ Compare DRL with heuristic − Did not compare learning time of DRL
[104]	Defect inspection problem in production line	CNN	Online		✓		✓		+ Distributed assignment with Lie Group − Offloading decision criteria not included
[105]	Addressing high dimensional continuous action space	DDPG	Online		✓		✓		+ Optimal offloading decision on edge − Left dependency aware task offloading
[106]	Minimize the service latency	DNN	Online	✓	✓		✓		+ Minimize end-to-end offloading delay − Critic and actor affect performance
[96]	Minimizing long-term system energy consumption	MADQN	Online		✓	✓			+ Intelligent updating of computing utilities − No result comparison with previous works
[91]	Designing efficient task offloading	DQL	Online		✓		✓		+ Consider reliability in time-varying topology − Centralized offloading decision
[94]	An optimal offloading decision	DQL	Online		✓		✓		+ Offloading using task size and queue − Searching queue space affect performance
[107]	Computation offloading and resource allocation	DDPG	Online		✓		✓		+ Considering time-varying network − Training delay registered
[59]	Minimize task completion time and energy consumption	DNN	Online		✓		✓		+ Analysis of obtaing accurate channel-state − Network resource analysis only
[57]	Maximize accuracy	CNN	Online		✓		✓		+ Consider trade-of low latency and energy − No offloading control strategy
[41]	Enhance offloading decision	MATD3	Online	✓	✓		✓		+ Introduce ratio-based offloading approach − Left long waiting delay
[37]	To minimize the latency and energy energy consumption	TD3	Online	-	✓	✓	✓	✓	+ Considering dynamic traffic loads − Latency problem on hotspot traffic scenario
[51]	Application execution delay	A3C	Online	✓	✓		✓		+ Comparison of offloading cost − Computation cost not discussed at fog node
[55]	Design optimal offloading decision policy	A3C	Online		✓	✓			+ Propose continual online learning − Search space grow performance issue
[63]	Balance learning accuracy and energy consumption	DDPG	Online	✓	✓			✓	+ Discuss task size and energy trade-off − Task offloading not covered

By selecting an appropriate training mode—offline, online, centralized, distributed, or federated—and leveraging the complementary strengths of DL and RL, DRL-based task offloading systems can combine the efficiency of pre-trained models with the adaptability required for real-world, dynamic fog computing environments. Each training paradigm presents trade-offs between latency, scalability, adaptability, and resource utilization, and the choice should align with the operational objectives, infrastructure capabilities, and privacy requirements of the intended deployment.

### 5.10. Application of DRL-Based Task Offloading in Fog Computing

DRL-based task offloading in fog computing networks enhances different applications while addressing their QoS requirements. Those applications benefited from the DRL-based fog computing architecture to handle real-time task processing with low latency demands. Some of the applications benefited from fog, smart transportation for tracking vehicles, optimizing routes, and managing peak time efficiently. In healthcare, DRL can optimize the processing of medical data generated by different smart healthcare devices, such as wearable health monitors. The wearable devices offload patient data to the fog node for processing in a real-time manner. By intelligently offloading tasks to fog nodes, healthcare providers can ensure critical patient data are processed quickly and efficiently, enabling real-time monitoring and timely interventions. This approach improves patient care while minimizing computational delay in data analysis. In smart city user devices, edge and fog nodes are integrated to manage utilities such as waste management, resource utilization, distribution, and lightning systems while improving decision making with DRL algorithms. DRL-based offloading also optimizes power distributions, and demand management can be facilitated with fog-based smart grid ecosystems. Applying DRL-based offloading in smart industries enables the fog paradigm to minimize task processing delay and increase productivity in manufacturing pipelines. Therefore, in industrial automation, the allocation of computational resources across fog nodes can improve operational efficiency, reduce downtime, and improve predictive maintenance strategies. Table 6 presents some of the applications that use task offloading supported by fog computing architectures.

### 5.11. DRL Agent Offloading Decision Criteria

DRL algorithms enhance the user device and consumer electronics task offloading decisions to different types of applications based on QoS requirements. The goal of this decision is to select the best fog node based on the QoS parameters to offload its task. The DRL agent uses criteria to make offloading decisions and maximize cumulative reward.

Task characteristics such as compute size, priority, and tolerable delay [112]; load information (computational capacity and task queue length); available energy/battery level; local computing capability and channel gains/conditions [107], network condition [112]; offloading mode (local, fog, and cloud) [65,113]; action space (discrete (binary) [107]; continuous (ratio or probability) and hybrid [40]; CPU cycles/frequency), communication resources (e.g., bandwidth, power allocation) to allocate for the task, either locally or at the offloading destination [42]; and previous experiences (replay memory storing past states, actions, rewards, and next states) are used to train the agent and inform decisions [41]; and device local computation capabilities [47].

- Types of tasks: The architectural nature of an end-user device network is to execute real-time tasks and generate real-time data. In this context, the problem is how to optimize QoS objectives such as response time and energy consumption. For example, in a delay-sensitive context, task completion time becomes a major criterion while offloading tasks. For delay tolerance tasks, offloading decision criteria might be varied based on the problem to be addressed.
- Available energy level: Mainly, one of the criteria from the end user device perspective is task offloading decisions made based on the available energy level of the fog node. For instance, the offloaded tasks are executed to maximize the remaining energy from the fog node by estimating the sum of computation and communication energy consumption [91].
- Time-slot: a specified time designed as a change in the balance between transmission time and execution time, with the complete response time the fog node takes.
- Bandwidth: the availability of the bandwidth concerning the latency requirement of the application to be offloaded leads to optimized decisions.
- Deadline: deals with guaranteeing the resource in the fog nodes to accomplish a task up to its lifetime with the current state of scheduled time and bandwidth.
- CPU Cycles and Task Size: The computation task of an end-user device is characterized by two key parameters: the number of required CPU cycles and the data size, whether for local processing or offloading. These parameters enable each device to make informed task-processing decisions based on both local system state and global system state information.
- Channel condition and end user device features: location, priority, satisfaction degree, and computational task are metrics to offload tasks to the fog node.

### 5.12. Model of Network Settings and DRL Agent Coordination Approaches

In DRL-based device—fog computing networks, the topological and computational structure plays a vital role in achieving QoS objectives. Equally important is the role of intelligent agents, which are responsible for decision-making, resource allocation, and information sharing across the network. This section explores these core concepts in detail, highlighting their significance in the design and performance of DRL-driven offloading systems.

## 6. DRL for QoS Optimization Strategies

This section presents a DRL-based approach to task offloading and resource allocation in fog computing, taking into account resource types, QoS objectives, and relevant QoS metrics. Several survey studies have examined the optimization of task offloading across user devices, edge, and fog environments, focusing on factors such as resource availability, the number of QoS objectives, and performance metrics. Broadly, these optimization strategies are categorized into mono-objective and multi-objective approaches, each aiming to satisfy specific QoS requirements under varying system constraints.

Table 6 discusses resource optimization with its objective functions and the pros and cons of each work. This table shows the relationship between task offloading and resource allocation in fog computing network settings for different applications. This table demonstrates that optimizing CPU resources is given high attention in the literature. Table 7 discusses resource optimization with its objective functions and the pros and cons of each work. This table shows the relationship between task offloading and resource allocation in fog computing network settings for different applications. This table demonstrates that optimizing CPU resources has received a lot of attention in the literature.

### 6.1. Resource Allocation and Task Assignment Approaches

Fog-based task offloading follows decentralized or centralized management approaches that can be applied to communication and task execution processes. From this perspective, the coordination of task offloading processes to assign a task and allocate resources for that specific task needs dynamic approaches. Both conventional and learning approaches are explored as a solution in the literature. In this regard, fog faces different challenges due to node mobility, heterogeneous resources, and limited resources. Moreover, most of the research works outline that allocating those limited and dynamic fog resources optimally is a fundamental challenge to execute tasks at the fog node while achieving QoS objectives. The challenge of managing fog resources becomes increasingly complex as the number of offloading choices grows with the number of fog devices, as discussed in [98].

Furthermore, the other core issue focuses on how to guarantee QoS requirements while allocating resources to tasks sent from user devices in this variable context. In support of this, Vu et al. [116] introduced the concept of task-to-server offloading relationships, including multi-subtask to multi-server offloading schemes, while addressing both task offloading and resource allocation challenges. Similarly, Salaht et al. [21] provided a comprehensive survey on service placement taxonomy, categorizing it as static or dynamic, offline or online, centralized or distributed, and either supporting or not supporting mobility. In the same vein, Apat et al. [117] conducted a survey on service-task placement algorithms aimed at minimizing (i) energy consumption, (ii) communication cost, (iii) latency, and (iv) load imbalance and maximizing QoS requirements.

A common concern raised by both studies is the reliance on conventional optimization methods for offloading management models, with limited exploration into the application of learning-based algorithms. In such traditional approaches, maintaining up-to-date fog resource information is essential but introduces performance bottlenecks. This continuous need for information gathering and updating can hinder the effectiveness of task offloading and execution, especially under strict QoS constraints. To conclude, knowing the statistics of fog nodes such as workload, resource (CPU size, storage, power), voluntary collaboration, task execution history, current status (active/disconnected), etc., is an essential parameter for both offloading and resource allocation decisions.

Table 8 highlights the types of resources investigated from the literature to optimize from different perspectives, and the data demonstrate that optimizing CPU resources is the main focus. The ✓ and − show the focus of the resource in the research work and are not included in that particular work, respectively.

#### Single vs. Multiple Resource Optimization

In a fog-based task offloading architecture, nodes are constrained with resources such as computing power, storage, bandwidth, battery, and channel conditions. Various studies have been conducted so far to optimize resource requirements for single and multiple resources to achieve QoS objectives. These QoS requirements are generated from the user device according to the types of application and its actual context. From a single resource perspective, Hou et al. [74] focused on optimizing battery life in task allocation problems while minimizing energy consumption for the swarm of drones to achieve reliability and latency. From the existing literature that considered multi-resource optimization, we quote the work of Jiang et al. [118] that focuses on optimizing bandwidth and battery level resources for deadline-driven applications to achieve a better trade-off between task response and device life span. Tang et al. [99] presented a decentralized computation offloading approach considering the computing power, buffer size, and battery life of the device. Finally, this work explores the opportunities of applying different DRL algorithms to correlate the task QoS requirement and resource availability in fog environments.

## 7. Future Research Directions and Open Issues

Applying AI/ML at the edge and fog has become a common practice to process real-time applications critical to delay in the industry. It enhances execution time by coordinating task processing between the user device and the fog nodes for task offloading decisions and resource allocation problems. Despite the promise of AI/ML, mainly DL, RL, DRL, and FL, for task offloading and resource allocation in fog computing, several key challenges and open issues need to be addressed to fully realize their potential. Specifically, the area of preserving security and privacy while offloading and smart offloading for emerging technologies needs to be addressed in fog computing networks.

### 7.1. Effective Management

A central challenge in this task offloading involves determining the most effective management architecture for the offloading process—whether it should be handled centrally by a single control server or distributed across multiple fog nodes. Centralized approaches may offer simplified coordination, but they can also introduce bottlenecks and single points of failure. In contrast, distributed management can enhance scalability and fault tolerance but may involve more complex coordination mechanisms. Another critical issue is the mobility of task-executing devices in dynamic and heterogeneous network environments. For instance, in scenarios involving unmanned aerial vehicles (UAVs) or mobile users, maintaining consistent connectivity becomes challenging. If the task execution time exceeds the duration of the device’s presence within the network range, offloading may fail or lead to incomplete processing, resulting in service disruption. Additionally, many existing task offloading strategies depend on static rules or predefined thresholds, such as fixed latency or energy consumption limits, to make decisions. While these methods offer simplicity, they often lack the flexibility to respond effectively to the highly dynamic and resource-constrained nature of fog environments. As a result, there is a growing interest in more adaptive, intelligent offloading mechanisms that can learn from changing conditions and make real-time decisions accordingly.

### 7.2. Role of Fog Computing and Smart Offloading Techniques for Emerging Technologies

Currently, the thinking of applying real-world scenarios is shifting from a traditional perspective of the physical world to the virtual world. In addition, fog computing brings a vast opportunity to provide latency-critical real-time applications with a smart offloading approach. Similarly, edge–fog-based smart offloading facilitates emerging technologies such as XAI, Metaverse, and ZeroTouch Network(ZTN) to make it a reality.

#### 7.2.1. Explainable AI (XAI)

In the AI/ML model, the decision-making process remains a black box, which means that the transparency attributes that determine the decision are not known. The question is “how does the model work to get particular outcomes?” and to interpret the output performance. Sarwar Murshed et al. [119] mentioned XAI on the network edge to add interpretation and explainable features to AI/ML models for training and decision-making. Likewise, knowing the weight of features allows for optimizing the offloading process for smart real-time service. Wang et al. [120] discussed the importance of XAI for emerging 6G technologies (ZTN and intelligent radio) and 6G use cases in Industry 5.0, emphasizing its role in providing transparency in the fast-speed decision-making process. For data-intensive IoT-Fog environments, explaining offloading decision parameters and prediction is critical. By highlighting the correlation between the model and its parameters, the result indicates which part of the model requires optimization. Therefore, XAI allows for the optimization of task offloading in wireless communication in terms of feature importance, feature correlation, and data interoperability. Additionally, more work is needed to design a technique that locally approximates the offloading model around a specific decision point, providing an interpretable explanation.

#### 7.2.2. Metaverse

A Metaverse is a 3D virtual space where the 2D virtual element and physical world are mapped to represent different experiences to create a shared digital environment [121]. The author states that objects interact to build a connected world when they react and reside in both physical and virtual contexts. A Metaverse is a shared continuous experience to make different activities together, rather than being constrained in one application. A typical example of a Metaverse is an augmented reality (AR) and virtual reality (VR) application that needs ultra-low latency response time. Different studies show that the Metaverse lacks a clear architectural design for those latency-critical applications. From this perspective, edge and fog architecture is a building block to enable the Metaverse technology to happen in reality [121,122]. A Metaverse with edge and fog computing brings unparalleled applications together and true support for mobility and borderless conditions. Currently, the Metaverse is not applicable at a full scale yet; the capability of the edge and fog device allows it to happen in reality, for instance, to handle high requirements from the latency-critical application. However, running a service in the Metaverse with fog needs an optimized resource sharing mechanism. Additionally, the challenge that comes from using computing power for shared activity leads to identity theft, real-life inequality, etc., which are areas that need to be addressed in the Metaverse.

#### 7.2.3. ZeroTouch Network

The Zero Touch Network (ZTN) avoids manual configuration and provides an easy plug-and-play environment for real-time service management. This concept is presented in different contexts, such as Zero Touch Network [123], Zero Touch Provisioning (ZTP) [124], and Zero Touch Management (ZTM) [125] for dynamic real-time application service provision. For this survey, the term ZTN is used for common understanding. Tehrani et al. [126] explain the application of machine learning algorithms for ZTN as an auto-scale, fast data-driven adaptation to different operating conditions. This is mainly important for task offloading from IoT devices to fog nodes by avoiding the delay that comes from configuration challenges. Therefore, it enhances latency-critical service provisioning through an auto-scaling approach by avoiding human-made manual configuration. However, configuration offloading is insecure since the IP assigned from DHCP is not authenticated, so requests and responses in the ZTN script need to be secured. Grasso et al. [125] and Alameddine et al. [127] proposed ZTN to solve offloading decisions for time-varying dynamic systems and real-data processing without considering previous network history. Plus, the ability of ZTN policy allows delaying sensitive applications to identify critical states to make the offloading decision and to scale resources [127]. Rezazadeh et al. [123] designed an AI-driven ZTN for a demand-aware resource allocation mechanism for a multi-objective 5G radio access network. The concern here is addressing the ZTN security problem while validating client and server artifacts before offering certificates to offload and execute tasks.

#### 7.2.4. Digital Twins

Digital Twins (DTs) are representations of physical assets and systems with virtual replicas that allow for real-time monitoring and control. The DT mainly advances with the advent of edge computing and Internet of Things (IoT) devices and has gained significant attention in various industries, including manufacturing, healthcare, transportation, and energy [35,128,129,130]. In this network environment, different optimization issues such as reducing latency, enhancing the performance of offloading decisions, and managing resource allocations are addressed. In this case, the DT provides the virtual representations of fog nodes to simulate the stochastic states of the node in the task offloading game. The application DT is demonstrated in different domains such as air–ground networks for seamless connection and real-time service and in industrial IoT [128], for military field, intelligent offloading, and resource allocation for smart industry [130], for the simulation of computation offloading with service caching in intelligent transportation [131], and others. However, dynamic resource management in continuously updating the DT with real-time data from real IoT–fog environments still needs further studies. It needs an optimized design and communication protocol to dynamically adjust resource allocations based on current demand, device availability, and network conditions.

#### 7.2.5. TinyML

Traditionally, user devices rely on edge, fog, and cloud environments to offload their tasks to meet the computational demands of various applications. However, when the user device offloads its data to the fog node, it incurs significant transportation and communication costs. In addition, offloading private data to unknown nodes raises security concern. To address these issues, the concept of Tiny Machine Learning (TinyML) ecosystems has recently emerged. In this approach, ML models are trained centrally, and the trained model is then deployed at the edge on the user device. Sanchez-Iborra and Skarmeta [132] discuss the application of TinyML for human lives, and a survey by Abadade et al. [133] also presents the application of TinyML for different domains such as precision agriculture, disaster risk management, smart appliances, optimizing energy utilization, wearable health trackers, waste management, smart traffic management, and many more. Even though ML models can be deployed to tiny user devices such as Raspberry Pi, Arduino, mobile phones, and others, they still have tiny memory resources and hardware face challenges in holding the model. This leads to the creation of TinyML frameworks such as Edge Impulse, Tensor Lite, MicroTVM, TinyML, TFLite micro, and others, training the model centrally and deploying it to large-scale user devices. In this case, continuously updating the model in the user device, as well as compatibility between those frameworks, needs further investigation.

### 7.3. Challenges in Multi-Agent DRL (MADRL)

MADRL is an extension of reinforcement learning that enables multiple agents to learn optimal policies through direct interaction with uncertain and dynamic environments [36]. A key strength of MADRL lies in its capacity to accelerate policy learning for complex tasks via parallel execution, which is particularly effective in cooperative and distributed systems such as fog computing [134,135]. However, coordinating multiple autonomous agents introduces several inherent challenges that significantly complicate policy optimization.

A primary difficulty is environment non-stationarity [136]. In a multi-agent system, the agent’s environment changes as other agents update their strategies, violating the stationary environment assumption underpinning most single-agent RL algorithms. This continual policy drift makes it harder for agents to converge toward stable, optimal solutions. Another critical issue is the credit assignment problem, where determining which agent’s actions contributed most to a team’s success or failure is non-trivial [137]. In cooperative scenarios, improper reward allocation can hinder effective policy updates, especially in sparse-reward environments. Scalability also presents a substantial challenge. As the number of agents grows, the joint state-action space increases exponentially—known as the curse of dimensionality—making centralized learning computationally prohibitive [138]. Similarly, many real-world applications shows partial observability, where agents have only local information about the system state [139]. This limitation forces agents to reason about hidden variables and infer the intentions of other agents, increasing decision-making complexity. Finally, communication and coordination remain open research problems. Designing protocols that enable agents to exchange information efficiently without incurring excessive bandwidth or processing overhead is critical [140].

Overall, agents’ poor communication can lead to redundant, conflicting, or suboptimal actions, reducing overall system performance. Addressing these challenges requires advanced strategies such as centralized training with decentralized execution (CTDE), curriculum learning, and attention-based communication mechanisms, which have shown promise in mitigating non-stationarity, improving credit assignment, and enhancing cooperation in large-scale MADRL systems [141,142].

### 7.4. Open Issues and Challenges

Task offloading approaches in the fog computing environment face two-dimensional challenges. The first challenge is the resource constraints of fog nodes, such as energy, computing power, and storage. Even though fog computing is relatively resource-rich compared to IoT devices, the resource competition problem still requires an optimized solution for task offloading and computation from IoT devices to fog nodes. Second, the heterogeneity and distributed nature of fog nodes, combined with a variety of task QoS requirements, present a challenge in resource allocation. Managing heterogeneous data generated by end devices, such as IoT sensors, smart CE, and actuators, is a major challenging issue in fog computing architecture. Moreover, in both cases, the problem is to balance task offloading decisions and the resource allocation approach from a resource-constrained device to resource-rich devices in the fog.

- QoS requirements: Optimizing offloading delay has been explored for real-time task processing in fog computing architecture. However, it is still required for the trade-off between various QoS requirements. In real-world task offloading between smart user devices, edge, and fog environments, energy, latency, and reliability are directly correlated; their relationship is not explored sufficiently at the required level.
- Resource utilization: In resource-constrained IoT-Fog environments, effectively utilizing these scarce resources is still a crucial challenge. This requires extracting and modeling dynamic network resources and providing load status impact on executing real-time tasks while achieving QoS requirements.
- Dynamic assignment: Coordinating task offloading and resource allocation in fog computing is unpredictably changing status; for this, a dynamic strategy is required. For task offloading decisions and resource assignment issues, [88] and [90] studied a dynamic network topology, and dynamic computation suggests that more work is needed, especially when the action space is continuous.
- Security and privacy-preserving offloading: Private data are generated from individual user devices and offloaded for processing to edge and fog nodes. However, maintaining security is difficult when sending confidential private information to an unidentified node for computation. Further investigation is needed to implement and design an intelligence security mechanism to protect the disclosure of private data for training and testing.

## 8. Conclusions

The fog computing paradigm is designed to address the computational challenges of delay-sensitive applications generated by the user devices. For such user devices, tasks are offloaded to fog nodes to solve the resource constraints problem to meet their performance requirements. Task offloading happens when local task computation models can not address the user device’s QoS requirements. Therefore, offloading to distributed fog nodes, depending on the goals of the applications, becomes a solution. A complete survey is presented on task offloading strategies that maintain task QoS requirements for real-time applications in the user device-to-fog computing environment. Plus, the application of the offloading model to different application domains in a real-time manner is investigated. As a result, various task offloading strategies in fog using centralized, decentralized (distributed), and federated architectural design approaches are presented. Specifically, techniques are presented for optimizing QoS requirements in task offloading for dynamic and static topological fog architectures. Moreover, different task offloading approaches emphasize the convergence problem, as the search space grows exponentially and increases run-time processing. Furthermore, applying continuous updating is challenging in these approaches because they focus on immediate results, which leads to overall performance degradation in the long run. Recently, RL techniques were utilized for this continuous action space problem, but there was a lack of historical knowledge of offloading decisions. Therefore, this work focuses on the application of DRL techniques to enhance task offloading and resource allocation decisions in the continuous search space of the fog. We also introduce a classification taxonomy in DL, RL, and DRL for the stochastic continuous search space with training and decision methods explicitly reported as online or offline decision-making techniques. Finally, potential study areas, open concerns, and challenges are presented in detail.

## Figures and Tables

**Table 2:** RQs with their motivation.

RQ	Description	Motivations
RQ1	In which scope does task offloading occur (user device to fog, edge to fog, fog to fog, or fog to cloud)?	To identify collaboration between multiple end devices and fog servers, as well as the corresponding use case scenarios.
RQ2	What are the types of applications that need a real-time offloading scheme?	To study different types of applications such as delay sensitivity and delay tolerance
RQ3	Which architecture management model used?	To demonstrate the concept of centralized, distributed, and federated approaches for task offloading problems.
RQ4	What QoS metrics are considered in the literature while offloading the task to the fog node?	To justify QoS requirement tradeoff such as energy, delay, and fault tolerance.
RQ5	Which DRL algorithms are commonly used in task offloading to decide where to compute tasks? Why?	To explore the existing learning algorithm to deal with task offloading problems in time-variant Fog computing networks.
RQ6	How should an offloading parameter be selected?	To identify determinant features and their degree of relevance.
RQ7	What are the future research directions and challenges?	To identify open issues of task offloading in fog.

**Table 3:** Categorizing task offloading approach based on agent action space.

Action Space	Reference
Discrete	[10,42,43,44,45,46,47]
Continuous	[34,38,48,49,50,51,52]
Hybrid	[40,53]

**Table 4:** Common DRL algorithms used for task offloading.

DRL Algorithms	Methods	Reference
Deep Q-Networks (DQN)	Value-Based	[42,44,49,52,88,89]
Proximal Policy Optimization (PPO)	Actor–Critic	[7,61]
Asynchronous Advantage Actor-Critic (A3C)	Policy Based	[40,45,53,55,68]
Deep Deterministic Policy Gradient (DDPG)	Actor–Critic	[10,50,63,80,90,91,92,93]
Twin Delayed DDPG (TD3)	Actor–Critic	[37,38,41]

**Table 6:** Some representative application of DRL-based task offloading in fog.

Application Type	QoS Objectives	Description	Reference
Smart transportation	Minimizing latency and improving reliability	Traffic monitoring, choosing best route, locating people waiting for service, tracking cars, managing roads, and accidents	[92,108]
Smart cities	Scalability and nergy efficiency	Utility monitoring, smart home locks, smart cameras, smart light control, and smart doorbells	[61,62]
Smart healthcare	Low latency and high reliability	Real-time decisions, analysis of patients, and data gathering	[24,58,80,109]
Autonomous vehicle	Latency and reliability	Object recognition and path planning	[61,74,110]
Smart grid	Efficiently balancing dnergy distribution	Balancing the idle time, resource wastage and peak time demand, and grid status	[53]
Smart Industry	To maintain low latency, high throughput, and efficiency	Delay-sensitive task processing demand within pipeline	[49,57]
Disaster recovery	Low latency	Real- time response and context mapping	[7,90]
Smart supply chain	Enhancing task transmission and communication latency	Monitoring the resource distribution from the source to the end user	[24]
Security and surveillance	Security and reliability	Real-time detection, identifying suspicious activity, and tracking of objects	[10,111]

**Table 7:** Resource and QoS objective optimization.

Reference	Resource	Objectives	Pros(+) and Cons(−)
[88]	Bandwidth and CPU cycle	Minimize task offloading latency	+ Considering fog node and task size for decision − No comparison made with similar works
[90]	CPU, memory, and bandwidth	Minimize energy consumption and computation latency	+ Focus on system stability and task offloading ratio − Offloading relationship is many-to-one
[114]	Channel state and CPU	Minimize learning and training delay in FL	+ Deal with computation and communication resource − Trade-off cost analysis not covered
[99]	Battery, CPU, and buffer size	Maximize network performance	+ Analyze decentralized partially observable offloading − Offloading decision and state not covered
[60]	CPU cycle and time	Optimize offloading decision and resource allocation	+ Weighted cost for local and offloading computation − No network resource included
[54]	CPU and fog resource	Minimize network latency	+ Consider dynamic resource on time-varying fog − No resource utilization status report by adversarial
[95]	Energy, time slot, and CPU cycle	Minimizing the training delay	+ Compare centralized and federated learning − No comparison on training delay and size of resource
[94]	Queue space	Minimize the processing time and overloading probability	+ Overall traffic and resource load analysis − Model-free approach raises bias on exploration
[115]	Battery level and time slot	Reduces the energy consumption, latency, and task drop rate	+ Resource trade-off analysis − Offloading queue space not addressed
[59]	CPU and bandwidth	Minimizing task completion time and energy consumption	+ Considers network efficiency and user experience − No application type analysis

**Table 8:** Resource optimization.

Reference	CPU	Bandwidth	Storage	Energy	Channel State	Que space	Battery	Buffer
[88]	✓	✓	-	-	-	-	-	-
[48]	✓	✓	-	✓	-		✓	-
[90]	✓	✓	✓	-	-	-	-	-
[114]	✓	-	-	✓	✓		-	-
[99]	✓	-	-	-	-	-	✓	✓
[60]	✓	-	-	-	-	✓		-
[54]	✓	✓	-	-	✓	-	-	-
[95]	✓	-	-	✓	-	-	✓	-
[94]	-	-	-	-	-	✓		-
[115]	-	-	-	-		✓	✓	-
[59]	✓	✓	-	-	-	-	-	-
[7]	-	✓	✓	✓	✓	-	-	-
[52]	-	✓	-	✓		✓		-
[96]	✓	✓	✓	✓	✓	-	-	✓
[89]	✓	-	✓	✓	-	-	✓	✓

## Data Availability

Not applicable.
